# Diverse genetic contexts of HicA toxin domains propose a role in anti-phage defense

**DOI:** 10.1128/mbio.03293-23

**Published:** 2024-01-18

**Authors:** Kenn Gerdes

**Affiliations:** 1 Kenn Gerdes is an independent researcher with the residence, Voldmestergade, Copenhagen, Denmark; University of Utah, Salt Lake City, Utah, USA

**Keywords:** HicA, HicB, toxin, RNase, antitoxin, RelE, phage, defense

## Abstract

Toxin–antitoxin (TA) modules are prevalent in prokaryotic genomes, often in substantial numbers. For instance, the *Mycobacterium tuberculosis* genome alone harbors close to 100 TA modules, half of which belong to a singular type. Traditionally ascribed multiple biological roles, recent insights challenge these notions and instead indicate a predominant function in phage defense. TAs are often located within Defense Islands, genomic regions that encode various defense systems. The analysis of genes within Defense Islands has unveiled a wide array of systems, including TAs that serve in anti-phage defense. Prokaryotic cells are equipped with anti-phage Viperins that, analogous to their mammalian counterparts, inhibit viral RNA transcription. Additionally, bacterial Structural Maintenance of Chromosome (SMC) proteins combat plasmid intrusion by recognizing foreign DNA signatures. This study undertakes a comprehensive bioinformatics analysis of genetic elements encoding the HicA double-stranded RNA-binding domain, complemented by protein structure modeling. The HicA toxin domains are found in at least 14 distinct contexts and thus exhibit a remarkable genetic diversity. Traditional bicistronic TA operons represent eight of these contexts, while four are characterized by monocistronic operons encoding fused HicA domains. Two contexts involve *hicA* adjacent to genes that encode bacterial Viperins. Notably, genes encoding RelE toxins are also adjacent to Viperin genes in some instances. This configuration hints at a synergistic enhancement of Viperin-mediated anti-phage action by HicA and RelE toxins. The discovery of a HicA domain merged with an SMC domain is compelling, prompting further investigation into its potential roles.

**IMPORTANCE:**

Prokaryotic organisms harbor a multitude of toxin–antitoxin (TA) systems, which have long puzzled scientists as “genes in search for a function.” Recent scientific advancements have shed light on the primary role of TAs as anti-phage defense mechanisms. To gain an overview of TAs it is important to analyze their genetic contexts that can give hints on function and guide future experimental inquiries. This article describes a thorough bioinformatics examination of genes encoding the HicA toxin domain, revealing its presence in no fewer than 14 unique genetic arrangements. Some configurations notably align with anti-phage activities, underscoring potential roles in microbial immunity. These insights robustly reinforce the hypothesis that HicA toxins are integral components of the prokaryotic anti-phage defense repertoire. The elucidation of these genetic contexts not only advances our understanding of TAs but also contributes to a paradigm shift in how we perceive their functionality within the microbial world.

## INTRODUCTION

Prokaryotes and their mobile genetic elements, such as phages and plasmids, have been locked in a co-evolutionary arms race spanning billions of years. In this intricate interplay, bacteria, and archaea have developed an arsenal of innate and acquired defenses against phages and plasmids. Acquired immunity, typified by CRISPR-Cas systems, relies on the memory of prior phage encounters. In contrast, innate defenses, like restriction-modification systems, are hardwired to indiscriminately degrade invasive genetic material. The concept of “Defense Islands” has been pivotal in recent discoveries by uncovering genomic regions in which defense genes are clustered (1–3). These revelations have unveiled a diverse array of defense strategies, demonstrating evolutionary links between prokaryotic and mammalian innate immunity systems. For instance, Theoresis phage defense systems possess domains akin to Toll-like receptors, and prokaryotic Viperins (pVips), like their mammalian orthologues, inhibit phage transcription by generating modified nucleotides (4). Similarly, anti-plasmid mechanisms employ Structural Maintenance of Chromosome (SMC) ATPases, which recognize signatures in foreign plasmid DNA and prevent plasmid establishment (1, 5, 6) (Robins WP, et al., 2023). Toxin–antitoxin (TA) modules, often situated in Defense Islands (1–3, 7), have only recently been associated with anti-phage activity, particularly via an abortive infection mechanism that halts infection at the cost of the host cell, thereby protecting the clonal population (8–11) (Smith, et al., 2023, in press). Recent investigations strongly support the notion that TAs commonly function as anti-phage elements (12–25).

TA modules are categorized into different types based on how the antitoxin counteracts the toxin. In Types I and III systems, small RNAs act as antitoxins by either blocking toxin translation or binding directly to the toxin, while Type II systems use protein antitoxins to achieve neutralization by direct protein–protein contact (26, 27). Based on toxin sequence similarity, the different types of TAs have been subdivided into families. For example, Type II TAs encode RelE, MazF, VapC, and HicA family toxins. All these toxins are RNases (also called mRNA interferases) that inhibit translation by cleavage of mRNA, rRNA, or tRNA and may thus induce abortive infection upon activation.

**Fig 1:**
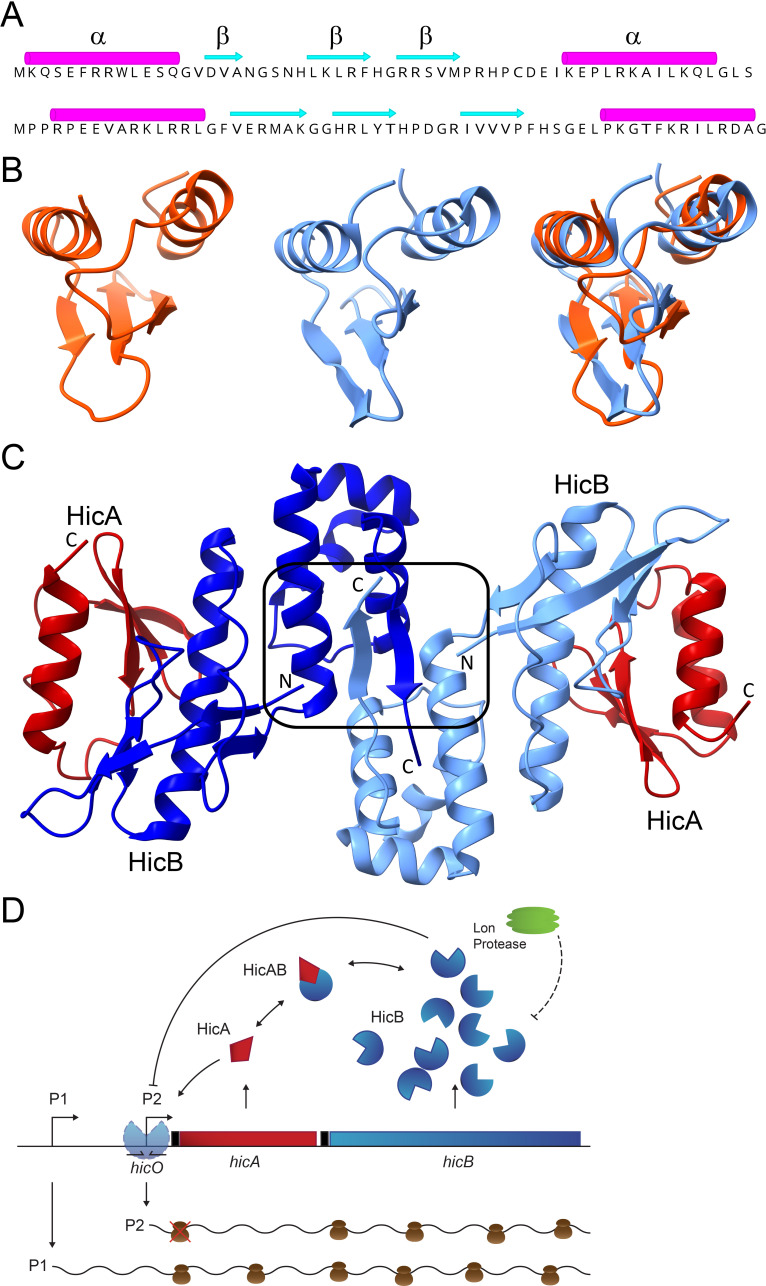
Components and regulatory features of the *E. coli hicAB* TA module. (**A**) Secondary structures of HicA of *E. coli* K-12 (upper) and HicA of *T. thermophilus* TTHA1913. (**B**) Tertiary structures of *E. coli* K-12 HicA (left, orange), *T. thermophilus* TTHA1913 HicA (middle, light blue), and their superimposition (right). In the superimposition of the two HicA structures, the root mean square deviation (RMSD) between 36 pruned atom pairs is 1.3 Å. The *E. coli* HicA structure was determined experimentally (33) while that of *T. thermophilus* was modeled by AlphaFold2. (**C**) Tertiary structure of the *E. coli* K-12 heterotetrameric HicA_2_B_2_ complex determined experimentally. The two HicAs are shown in red while the two HicBs are shown in blue and light blue. The HicB helix-turn-helix (HTH) DNA-binding motifs that dimerize HicB are boxed in. The figure was adapted from reference (33). (**D**) Proposed regulation of the *E. coli hicAB* operon (35). The *hicAB* operon is transcribed by two promoters, P1 and P2. During steady-state growth conditions, P1 is constitutive while P2 is repressed by HicB binding to the *hicO* operator. Excess HicA toxin ([HicA] > [HicB]) destabilizes HicBs binding to *hicA* and excess of HicA therefore leading to activation of P2. Notably, the *hicAB* transcript synthesized by P2 produces HicB but not HicA. Therefore, the HicA-mediated derepression of *hicAB* transcription specifically stimulates synthesis of HicB but not HicA under conditions of excess HicA. The *hicAB* operon of *Burkholderia pseudomallei* is regulated by a related mechanism: excess of HicA destabilizes the binding of HicB to the operator in the promoter region (34). Symbols: arrows indicate stimulation, and lines ending in a bar symbolize inhibition or protein degradation (Lon hexamer shown in green degrades HicB (28) shown in blue; HicA is shown in red). *hicO* symbolizes the inverted repeat to which HicB dimers bind and repress transcription. Messenger RNAs are shown as wavy lines and ribosomes as brown bodies. A red cross-over symbolizes that the *hicA* cistron of the P2-generated transcript is not translated.

HicA toxins constitute a large family of small, mono-domain RNases ranging from 50 to 100 amino acids. They are found in both bacterial and archaeal species, often in multiple copies per genome (28, 29). The core of HicA RNases exhibits the characteristic α-β-β-β-α topology of the double-strand RNA Binding Domain (dsRBD) fold (Fig. 1A) (29–34). Superimposition of the crystal structures of *Escherichia coli* K-12 HicA and HicA of *Thermus thermophilus* HB8 known to exhibit the dsRBD topology (29) exposes their highly related tertiary structures (Fig. 1B).

The HicA toxins that have been examined experimentally are all encoded by bicistronic *hicAB* operons where the downstream gene encodes a HicB antitoxin. In these systems, HicB comprises an N-terminal partial RNase H domain and a C-terminal DNA-binding-domain (DBD) of the helix-turn-helix (HTH) or the ribbon-helix-helix (RHH) type (29, 33, 34). The partial RNase H domain of HicB exhibits a β-β-β-α-β-α topology, with the first four secondary structure elements characteristic of partial RNase H folds (Fig. S1A) (29). The superimposition of *E. coli* K-12 HicB’s partial RNase H fold on that of TTHA1013 from *T. thermophilus* HB8, known to possess a partial RNase H fold, confirms the similarity of HicB’s fold (Fig. S1B).

Crystallographically, HicA and HicB of *E. coli* K-12 form an A_2_B_2_ heterotetrameric complex (33) (Fig. 1C). HicB interacts with HicA by packing of helix α1 of the partial RNase H motif against the β sheet of HicA (Fig. 1C), mirroring the observed behavior in RHH-containing HicAB complexes *of Burkholderia pseudomallei* and *Streptococcus pneumoniae* (32, 34). This implies that HicA inhibition by HicB operates independently of the type of HicB DNA-binding domain.

HicB binds to palindromic operators in the *hicAB* operon promoter region via its C-terminal DBD, thereby autorepressing transcription (Fig. 1D). Notably, high levels of HicA destabilize the HicAB-DNA complex and thereby stimulates operon transcription (34, 35). This phenomenon, observed in many other type II TA families, underscores the intricate repression and derepression mechanisms of *hicAB* operons (36–39). Further details of *hicAB* operon regulation and derepression are described and visualized in Fig. 1D.

Motivated by the exponential growth of microbial DNA databases, this study undertakes a comprehensive bioinformatics analysis of genetic elements encoding HicA dsRBD domains. The findings reveal the presence of HicA domains in at least 14 distinct genetic contexts, eight of which adhere to the canonical bicistronic TA operon configuration. Remarkably, only two of these genetic contexts have undergone experimental analysis. Four configurations encompass monocistronic operons, featuring fused HicAB domains, while the remaining two configurations involve *hicA* genes in operon with bacterial Viperins, which serve as guardians against bacteriophage invasions (4). The most common bicistronic operon structure is *hicBA* in which antitoxin HicB does not have a DBD, raising the question of how transcription of these operons is regulated. Lastly, the discovery of a HicA domain fused to a SMC domain raises intriguing functional questions.

## RESULTS AND DISCUSSION

### Fourteen classes of HicA domains

**Fig 2:**
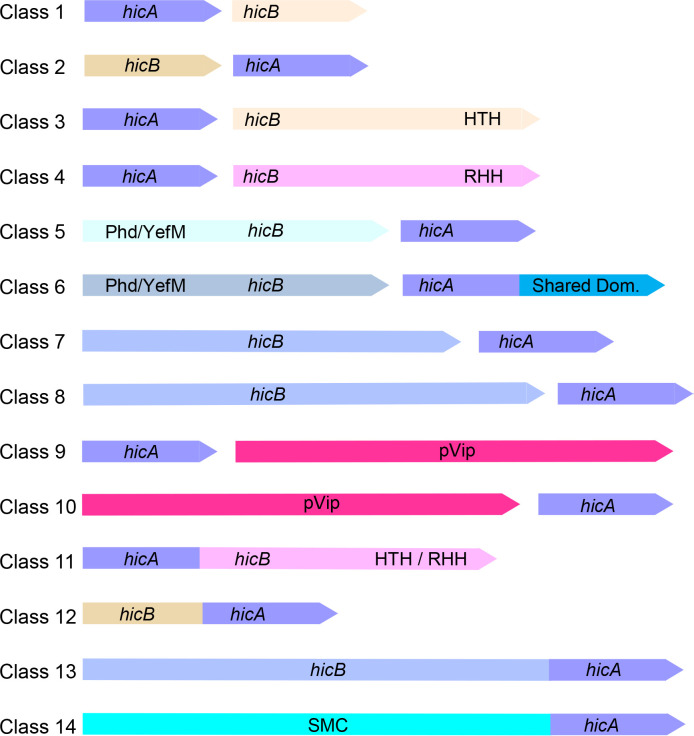
Fourteen distinct genetic contexts encoding HicA domains. Genetic organization of 14 HicA-domain classes derived from data in Table S1 (Sheets 1–14). Class 1: *hicAB* in which HicB is small (67–97 amino acids) and devoid of a recognizable DBD; Class 2: *hicBA* with a reversed gene order compared to that of Class 1 in which *hicB* also is small (63–103 codons) and devoid of a recognizable DBD; Class 3: *hicAB* in which HicB has a C-terminal HTH domain; Class 4: *hicAB* in which HicB has a C-terminal RHH domain; Class 5: *hicBA* with a reversed gene order compared to Classes 3 and 4 and in which *hicB* has an N-terminal Phd/YefM DBD; Class 6: similar to Class 5 but HicA has an extended C-terminal domain called the Shared Domain; Class 7: *hicBA* loci in which *hicB* is larger than *hicB* of the previous classes (216–233 codons); Class 8: *hicBA* loci in which *hicB* is even larger than *hicB* of Class 7 (331–356 codons); Class 9: *hicA* upstream of a gene encoding a pVip; Class 10: *hicA* downstream of a gene encoding a pVip; Class 11: *hicAB* monocistronic operon encoding a HicA domain fused to HicB with a C-terminal DBD (HTH or RHH); Class 12: *hicBA* monocistronic operon encoding a HicA domain fused to a small HicB domain; Class 13: *hicBA* monocistronic operon encoding a HicA domain fused to a large HicB domain; Class 14: A SMC domain fused to a C-terminal HicA domain.

Through database searches utilizing experimentally verified HicA toxin sequences, a striking diversity of genetic contexts encoding HicA domains emerged. Automated inspection of the neighboring sequences led to the classification of HicA domains into 14 distinct sequence classes (Fig. 2; Table S1). Each automated gene annotation was meticulously validated by manual examination of the DNA sequences. The 14 classes encompass a wide spectrum of genetic organizations, with Classes 1 to 10 featuring bicistronic operons, resembling the genetic arrangement of typical bicistronic TA modules. In contrast, Classes 11 to 13 consist of monogenic operons that encode fused HicA and HicB domains, while Class 14 is an interesting case of an SMC domain fused to a HicA domain. Notably, HicA-encoding genes exhibit a ubiquitous presence across prokaryotic phyla, underscoring their prevalence in the prokaryotic realm (Table S1). However, it is noteworthy that Classes 3 and 4 are relatively scarce in Archaea, and archaeal HicA-encoding TA loci often belong to Class 1 or 2. The following sections describe the distinctive features of these diverse classes.

### Classes 1 and 2: small and compact *hicAB* modules

Classes 1 and 2 comprise *hicAB* and *hicBA* operons, respectively, with HicBs encoded by these modules characterized by their diminutive size and the absence of an identifiable DBD in HicB (Fig. 2). Consequently, these TA modules are exceptionally compact, typically encoding HicAs of 60 to 85 amino acids and HicBs of 60 to 90 amino acids. In most instances, the *hicA* and *hicB* genes of both Classes 1 and 2 are closely linked or exhibit overlap, suggesting translational coupling (Table S1). The lack of DBDs of HicB is significant because it raises questions regarding the regulatory mechanisms governing the expression of the operons. It can be argued that Class 1 HicBs, devoid of a DBD, are non-functional genes arising from premature stop-codon mutations (Fig. 2). While database searches indeed uncovered instances of such cases, a thorough sorting of Class 1 HicBs using BLASTP analyses supports the functionality of most Class 1 *hicAB* modules, as explained in detail in the Materials and Methods.

**Fig 3:**
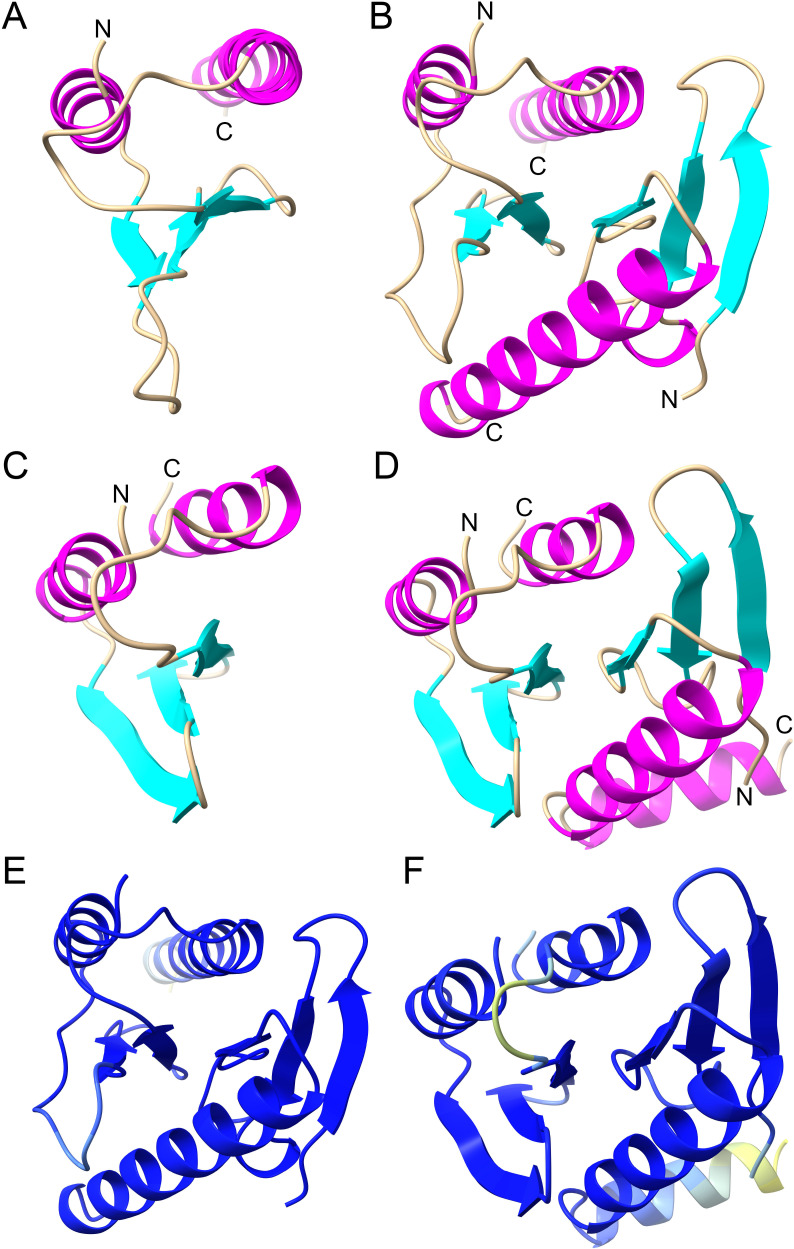
Modeling of Classes 1 and 2 HicA monomer and HicAB dimer structures. (**A**) Structure model of Class 1 HicA monomer of *Planktothrix* phage PaV-LD (YP_004957304.1) generated by AlphaFold2. (**B**) Structure model of Class 1 HicAB dimer of *Planktothrix* phage PaV-LD (YP_004957304.1, YP_004957303.1) generated by MultiFOLD. Dimer plDDT: 0.969; pTM: 0.903; Assembly quality: 0.9503; Interface quality: 0.9524. Assembly and Interface quality were calculated by ModFPOLDdock. (**C**) Structure model of Class 2 HicA monomer of *Campylobacter* sp. RM12654 (MBZ7977437.1) generated by AlphaFold2. (**D**) Structure model of Class 2 HicAB dimer of *Campylobacter* sp. RM12654 (MBZ7977437.1, MBZ7977438.1) generated by MultiFOLD. Dimer plDDT: 0.925; pTM: 0.850.; Assembly quality: 0.9125; Interface quality: 0.9069. Assembly and Interface quality were calculated by ModFOLDdock. (**E**) and (**F**) Dimer structure models from (**B**) and (**D**) colored according to the AlphaFold2 quality scheme (blue represents high quality, yellow low quality).

Notably, toxins and antitoxins of Classes 1 and 2 have not yet undergone experimental analysis. Structure modeling unveiled that Classes 1 and 2 HicA toxins from a cyanobacterial phage and *Klebsiella pneumoniae* feature the canonical α-β-β-β-α fold (Fig. 3A and C), as first reported for the HicA dsRBD (29). The corresponding HicA•HicB dimer structures are shown in Fig. 3B and D, respectively. Interestingly, phage-encoded HicA in complex with its cognate HicB is predicted to undergo a conformational change, potentially resulting in the loss of its N-terminal β-sheet (Fig. 3B). In contrast, HicA of *K. pneumoniae* does not exhibit such a change in configuration in the predicted complex (Fig. 3D). One possibility is that in the phage HicAB complex, HicA changes configuration and thereby loses its RNase activity—that is—HicA’s interaction with HicB leads to a structural change that inactivates the enzyme. Alternatively, the predicted structural change might be an artifact of the modeling process, necessitating further investigation to elucidate this issue. Importantly, both HicBs of the HicA•HicB complexes exhibits the canonical β-β-β-α-β configuration of the partial RNase H fold (29) (Fig. 3B and D). The AlphaFold2 modeling produced high-quality structures, further confirmed by ModFOLDdock analysis, yielding high Assembly and Interface quality scores (Fig. 3E and F).

### Classes 3 and 4: classical *hicAB* operons with HTH or RHH DNA-binding domains

Classes 3 and 4 encompass the model *hicAB* operons, with Class 3 encoding HicB antitoxins with a C-terminal HTH DBD and Class 4 HicB having a C-terminal RHH DBD (Fig. 2). As elaborated in the Materials and Methods, all DNA binding domains were rigorously validated using AlphaFold2, FoldSeek, Phyre2, or, in a few ambiguous cases, by sequence similarity searches (BLASTP). In experimentally analyzed modules, HicBs employ their HTH or RHH domains to autorepress transcription of their cognate *hicAB* operon by binding to palindromic operators in the promoter regions. Simultaneously, the antitoxins counteract the detrimental RNase activity of HicA (as illustrated in Fig. 1D). The distances between *hicA* and *hicB* in these operons vary: a substantial portion (31% of 119) of Class 3 genes are closely linked (with a separation of ≥10 bases between *hicA* and *hicB*; Table S1), and a similar trend is observed in Class 4 genes (41% of 171). Several experimental structures of the components encoded by Classes 3 and 4 operons have been elucidated and will not be explored further here (30–34).

### Classes 5 and 6: *hicBA* modules with unconventional arrangements

**Fig 4:**
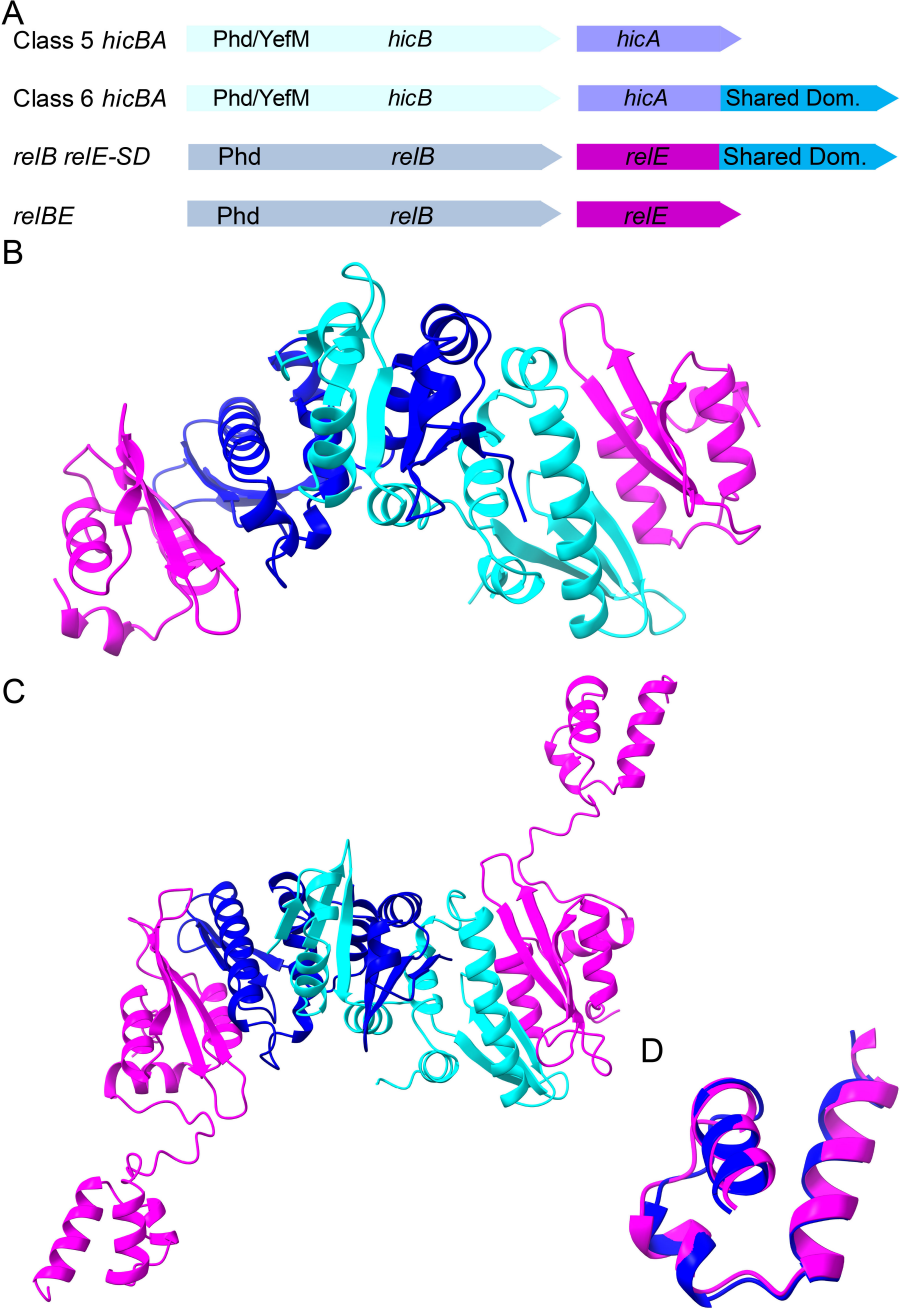
Genetic organizations and structures of Shared Domains. (**A**) Comparison of the genetic organizations of HicA and RelE toxins with Shared Domains. Classes 5 and 6 *hicA* are in operons with a upstream *hicB* encoding Phd/YefM DBDs. RelE with Shared Domains also has cognate antitoxins with Phd/YefM DBDs encoded by upstream *relB* genes. *relB* genes encoding Phd/YefM DBD in their N-terminal parts were identified using webFlaGs. (**B**) Structure of tetrameric Class 5 HicA_2_B_2_ complex (WP_078438722.1, WP_217700609.1). HicB dimerizes via the N-terminal Phd/YefM DBD and interacts with HicA monomers mainly via its central β-sheet of the partial RNase H-fold that aligns with α-helix 2 of HicA. (**C**) Structure of tetrameric Class 6 HicA_2_B_2_ complex (MBB5067807.1, MBB5067806.1). Again, HicB dimerizes via the N-terminal Phd/YefM DBD and interacts with HicA monomers mainly via its central β-sheet that aligns with α-helix 2 of HicA. The Shared Domains of the two HicA monomers are indicated with a green circle. (**D**) Superposition of Shared Domains of Class 6 HicA (MBB5067807.1) and a RelE toxin (WP_141355914.1) with a Shared Domain (From Fig. S4). The superposition has an RMSD of 0.838 Å across the entire structures (38 pairs).

Classes 5 and 6 represent a distinctive departure from the typical *hicBA* genetic organization. These classes feature a reversed gene order compared to the classical Classes 3 and 4 *hicBA* modules and encode HicB antitoxins with a Phd/YefM DBD in their N-termini (Fig. 2 and 4A). Similar to the HTH and RHH domains of HicBs of Classes 3 and 4, the Phd/YefM DBD of Classes 5 and 6 HicBs dimerizes the complex and likely function to regulate transcription of their cognate *hicBA* operons via binding to operators in the promoter regions (Fig. 4B and C). Most *hicBA* genes belonging to Classes 5 and 6 are closely linked and in many cases overlap, indicating translational coupling. Classes 5 and 6 introduce a novel feature of *hicBA* operon regulation by utilizing Phd/YefM DBDs.

HicAs from Classes 1 to 5 are small, mono-domain proteins featuring a dsRBD fold. However, Class 6 HicAs have an additional domain, approximately 55 to 60 amino acids in size, situated at their C-termini, referred to here as the “Shared Domain” (Fig. S2). Notably, conserved prolines found at the junction between the N-terminal HicA dsRBD domain and the Shared Domain may act as “domain-breakers” maintaining separation between the N and C-terminal domains (Fig. S2). While Class 6 HicAs are predominantly present in Actinomycetes, they also occur in other phyla.

BLAST analyses led to the identification of RelE/ParE toxins also consisting of two domains where the C-terminal domain exhibits sequence similarity to the Shared Domain of Class 6 HicAs. Sequence alignments of RelE/ParE toxins with and without a Shared Domain reveal a pattern very similar to that of the alignments of Classes 5 and 6 HicAs (Fig. S3). Alignment of the Shared Domains of Class 6 HicAs and RelE/ParE toxins demonstrate their sequence similarity and their conserved secondary structure (Fig. S4). The genetic organization of Class 6 *hicBA* and *relBE* with a Shared Domain are strikingly similar, suggesting a common function of the Shared Domains of the two toxin families (Fig. 4A).

The structural modeling of the tetrameric Class 5 HicA_2_B_2_ complex show that HicB dimerizes through the Phd/YefM domains of HicB by domain-swapping (Fig. 4B). The two HicAs interact solely via each HicB subunit and do not interact themselves. Hence, the Class 5 HicA_2_B_2_ complex exhibits a compact structure that in this respect resembles the crystal structure of the HicA_2_B_2_ complex of *E. coli* K-12 (33) (Fig. 1C). In the Class 6 HicA_2_B_2_ complex, the modeled structure remains largely similar, with the exception of the Shared Domain connected to the dsRBD of HicA through a long, flexible linker (Fig. 4C). Notably, the Shared Domain of Class 6 HicAs, consisting of three α-helices, extends outward from the compact structure and may thus be available for interactions with external factors.

A similar theme arises with RelB_2_E_2_ complexes in which RelE is extended by a C-terminal Shared Domain. As seen from Fig. S5A, the canonical RelB_2_E_2_ complex of *E. coli* K-12 exhibits a V-shaped structure generated by dimerization via the RHH domains of RelB and RelB domain-swapping (40). The RelE subunits interact with the C-terminal domain of RelB. A RelB_2_E_2_ complex in which RelE has a C-terminal Shared Domain complex in which two RelBs dimerize by domain-swapping via their Phd/YefM domains forming a structure in which the two RelEs interact with the C-terminal parts of RelB (Fig. S5B). Again, the Shared Domain, consisting of three α-helices, extends outward from the complex suggesting potential interactions with external factors. Superposition of the Shared Domains from a RelE and a Class 6 HicA reveals a remarkable root mean square deviation (RMSD) of 0.838 Å, indicating a common ancestral origin (Fig. 4D). The presence of domains shared across distinct toxin families is unique and deserves experimental scrutiny.

### Classes 7 and 8: remarkable diversity of *hicBA* modules

These classes are characterized by encoding relatively long HicB antitoxins, with Class 7 HicBs spanning 216 to 233 amino acids, and Class 8 HicBs extending from 342 to 356 amino acids (Fig. 2). In line with typical TA modules, the *hicA* and *hicB* genes within these classes are closely linked, and in many instances overlap (Table S1). For Class 7, structural modeling was employed to gain insight into the HicA and HicB components. The Class 7 HicA, encompassing a typical dsRBD, signifies its potential role as an RNase (Fig. S6A). On the other hand, Class 7 HicB comprises two domains separated by an α-helix (Fig. S6B). FoldSeek searches identified the presence of DUF1902 domains in both the N and C-terminal parts of HicB. DUF1902 domains are characterized by the presence of an α-helix and four β-strands. (Fig. S6B). Using webFlaGs, it became apparent that many DUF1902-encoding genes are juxtaposed to a *hicA* gene. This insight provides a potential avenue for further investigation into the significance of DUF1902 domains. A Class 7 HicAB dimer model further supported the notion that HicA likely interacts with the anti-parallel β-sheets of the C-terminal domain of HicB (Fig. S6C).

Class 8 introduces additional complexity, with a longer Class 8 HicA of ca. 95 amino acids when compared to canonical mono-domain HicA toxins (Classes 1 to 5). Class 8 HicA retains the characteristic α-β-β-β-α dsRBD fold but features two additional small α-helices at the C-terminus (Fig. S7A). Importantly, secondary structure predictions based on a sequence alignment of Class 8 HicAs revealed the conservation of these two α-helices (Fig. S7C).

Class 8 HicB exhibits even greater complexity, characterized by three distinct domains (Fig. S7B). The middle domain (aa 175 to 253) shares structural similarities with lysyl-tRNA synthetases, the C-terminal domain (aa 255 to 342) displays the HicB-fold, and the N-terminal domain (aa 1 to 171) does not exhibit significant similarity to domains with known functions. Alignment of HicB sequences confirmed the three-domain structure (Fig. S8). The structural similarities found in Class 8 HicB domains open avenues for further exploration, especially regarding the functional role of the middle domain that resembles lysyl-tRNA synthetases. Notably, neither HicA nor HicB of Classes 7 and 8 feature a recognizable DBD, again raising questions about how the synthesis of these proteins is regulated.

### Classes 9 and 10: *hicA* and *relE* are adjacent to prokaryotic Viperin genes

Eukaryotic Viperins are antiviral proteins that modify CTP and thereby cause termination of viral RNA synthesis (41, 42). In turn, infection by a broad range of RNA and DNA viruses is inhibited. Prokaryotic Viperins are orthologues of eukaryotic Viperins that have anti-phage activity (4). Notably, pVip antiviral activities extend beyond CTP to include the modification of GTP and UTP, making them powerful defenders against phage infections (4).

As also noted by others (4), *hicA* genes are found adjacent to genes encoding pVIPs, presenting a fascinating convergence (Fig. 2). Genes encoding HicAs are located both upstream (Class 9) and downstream (Class 10) of pVIP genes, in both cases adjacent to the pVIP-encoding gene (Table S1). The *hicA* genes located upstream and downstream of the pVip genes all exhibit the typical HicA secondary (α-β-β-β-α) and tertiary structures, suggesting that at least some of these HicAs are active toxins (Fig. S9).

A further fascinating revelation emerged during the exploration of *hicA* genes linked to pVIPs: RelE-encoding genes are also closely associated with pVIP-encoding genes (Fig. S10A). The RelEs that accompany pVIPs adhere to the typical RNase I fold characteristic of RelE-homologous RNases (Fig. S10B and C). This finding underscores the possibility that both HicA and RelE RNases operate in tandem with pVIPs to mount a robust defense against phage infections.

When activated, canonical HicA and RelE RNases arrest translation (28, 43, 44). In many cases, inhibition of translation by TA-encoded toxins contributes significantly to phage defense by facilitating abortive infection, a phenomenon observed with both Type 2 and Type 3 TA systems (10, 21, 45, 46) (and additional references op. cit.). The genetic linkage of pVips and two evolutionary independent RNase families raises several important questions, in particular, how is production and activity of the toxic RNases regulated and do the RNases contribute to the antiviral activity of pVips?

### Classes 11 to 13: distinct features of fused *hicAB* and *hicBA* modules

Classes 11 to 13 have a unique configuration where HicA and HicB domains are fused, forming monocistronic operons, a notable departure from the typical bicistronic structure in other classes (Fig. 2). Particularly, Class 11 HicBs have DNA-binding domains (HTH or RHH) at the C-termini, reminiscent of the HicBs of Classes 3 and 4 (Table S1).

**Fig 5:**
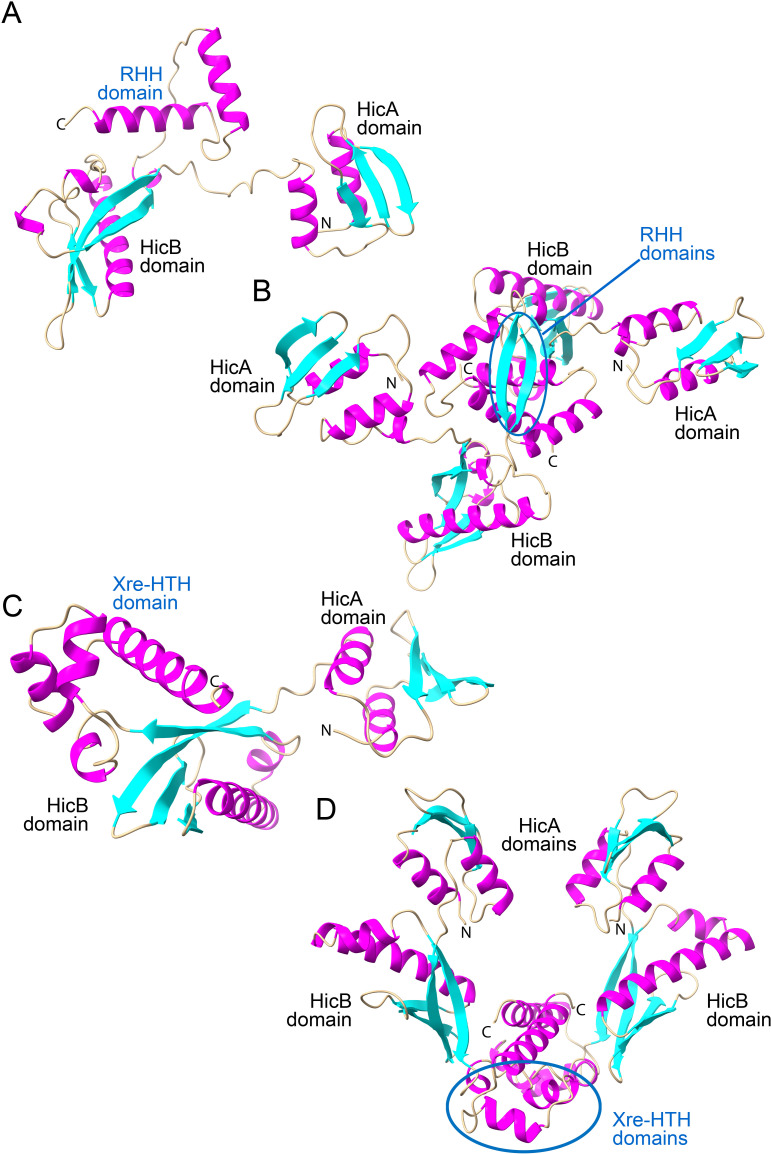
Structure models of Class 11 fused HicAB mono-domain TAs. (**A**) Monomer model of Class 11 fused HicAB (KPW96986.1) generated by AlphaFold2 with its three domains marked up. (**B**) Dimer model of Class 11 fused HicAB (KPW96986.1). The monomers dimerize via their RHH domains (blue ellipse). (**C**) Monomer model of Class 11 fused HicAB (WP_243550782.1). (**D**) Dimer model of Class 11 fused HicAB (WP_243550782.1). The monomers dimerize via their Xre-HTH domains (blue ellipse).

Molecular modeling shows that Class 11 HicBAs have three domains: a canonical N-terminal HicA domain, a middle RNase H domain, and a C-terminal DNA-binding domain (RHH or HTH) as illustrated in Fig. 5A and C. Both RHH and HTH domain-containing subclasses form dimers, a critical feature for DNA binding (Fig. 5B and D). The superposition of experimental RHH and HTH structures with Class 11 models, detailed in Fig. S11, supports this dimerization through DNA-binding domains.

**Fig 6:**
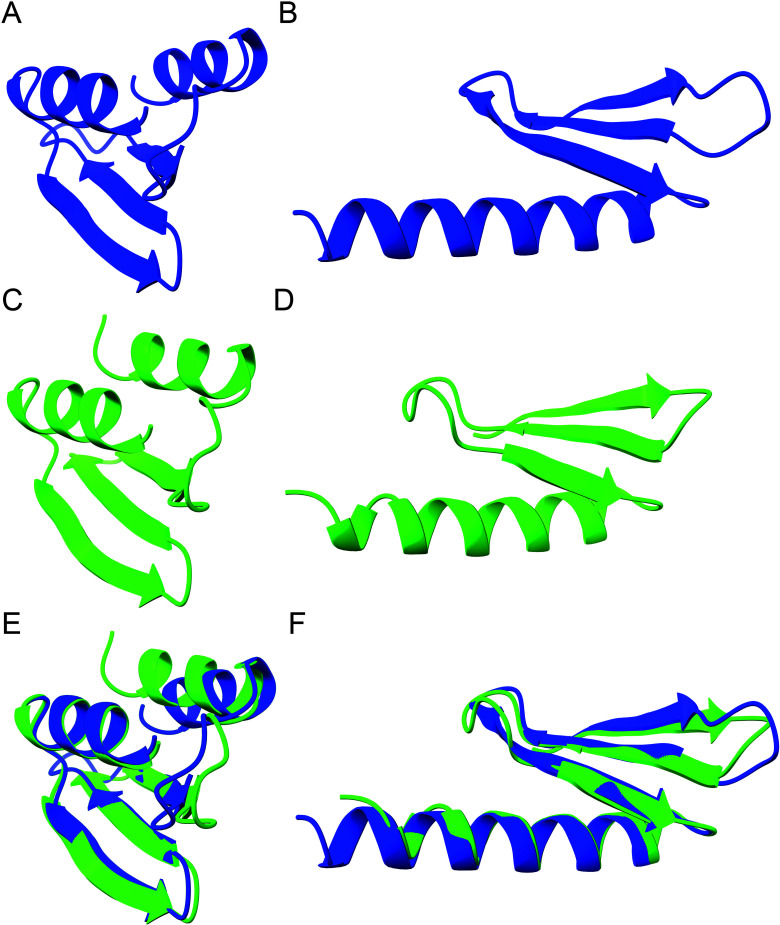
Comparison of Classes 2 and 12 HicBA structure models. (**A**) Class 12 HicA domain in blue (aa 74 to 127 of MBI3260764.1; #5 in Sheet 12 of Table S1). (**B**) Class 12 HicB domain in blue (aa 12 to 60 of MBI3260764.1; #5 in Sheet 12 of Table S1). (**C**) Class 2 HicA in green (aa 9 to 63 of MBZ7977437.1; #129 in Sheet 2 of Table S1). (**D**) Class 2 HicB in green (aa 2 to 46 of MBZ7977438.1; #129 of Sheet 2 in Table S1). (**E**) Superimposition of structures in (**A**) and (**C**) (RMSD between 48 pruned atom pairs is 0.907 Å). (**F**) Superimposition of structures in (**B**) and (**D**) (RMSD between 46 pruned atom pairs is 0.779 Å). All structure models were generated by AlphaFold2 and annotated in ChimeraX.

Fused Class 12 HicBA resemble Class 2 HicBA in terms of gene orientation and protein sizes (Fig. 2). This structural similarity is reinforced through molecular modeling, which reveals that the HicA domain of a Class 12 HicBA aligns remarkably well with a Class 2 HicA (Fig. 6A, C, and E), with a notable RMSD of only 0.907 Å. Similarly, the HicB domains of these classes align closely (RMSD of 0.779 Å), hinting at an evolutionary linkage ( Fig. 6B, D, and F). This suggests a potential evolutionary trajectory involving the fusion of ancestral separate *hicBA* genes, although the reverse scenario cannot be excluded.

The functional aspects of Class 12 HicBA were addressed using a phylogenetic analysis comparing artificially fused Class 2 HicBAs with naturally fused Class 12 HicBAs (Fig. S12). Notably, several Class 12 HicBAs cluster distinctly, indicating that the proteins evolve under selection pressure. This observation indicates that at least some of the Class 12 HicBAs are functional.

Class 13 HicBAs are larger (340 to 440 aa) and feature a unique domain arrangement with predominantly α-helices in the N-terminal region and a C-terminal domain following the canonical HicA configuration (Fig. S13A and B).

HicA belonging to Classes 11 to 13 all share the “fused” configuration and in this respect obviously departs from the canonical Type II TA paradigm. This unusual organization raises the question of how the toxin activity of the fused TAs is regulated. Importantly, in this connection, some fused Type II TAs have been investigated experimentally. The fused TA system CapRel^SJ46^ protects *E. coli* against diverse phages (15). Notably, the C-terminal domain of CapRel^SJ46^ regulates the toxic N-terminal region, serving as both an antitoxin and phage infection sensor. After infection by certain phages, newly synthesized major capsid protein of the phage binds directly to the C-terminal domain of CapRel^SJ46^ to relieve autoinhibition, enabling the toxin domain to pyrophosphorylate tRNAs, which blocks translation and thereby restricts viral infection. It is thus possible that the fused HicABs function by similar or related mechanisms to curb phage infection. In addition, the EzeT TA protein of enterobacterial strains consists of two domains in which the C-terminal domain is a toxic kinase while the N-terminal domain inhibits the kinase activity and thereby functions as a cis-acting antitoxin (47).

### Class 14: Novel fusion of SMC and HicA domains

Class 14 introduces a surprising fusion of SMC and HicA domains, a combination not previously observed in prokaryotic proteins (Fig. 2). Prokaryotic SMC proteins are large ATPases involved in chromosome organization and segregation (48, 49). As with other SMC-like proteins, substantial portions of the SMC-HicA proteins consist of α-helices that fold into coiled-coil structures (Fig. S14A and B). The Class 14 HicA domains exhibiting the canonical dsRBD configuration are fused to the C-terminal ends of the SMC domains (Fig. S14B and C). Interestingly, some of the SMC-HicA hybrid proteins also have a NERD-domain predicted to have nuclease activity and may function in DNA processing (50).

The function of the HicA domain fused to an SMC homolog remains enigmatic. However, recent discoveries have highlighted the roles of systems like Wadjet and Lamassu (also known as DdmABC in *Vibrio cholerae*) in defending against phage attack and plasmid transformation (1, 5, 6) (Robins WP, et al., 2023,). These systems encode SMC homologs that are essential for defense activities. The SMC homologs can be activated by specific signatures in incoming DNA elements. Depending on the system, they either degrade foreign DNA (WadJet) or induce abortive infection (Lamassu) upon detecting invasive elements (5, 6, 51) (Robins WP, et al., 2023). It is thus possible that the ribonuclease activity in Class 14 SMC-HicA proteins might similarly be triggered by foreign DNA or RNA, potentially playing a role in abortive infection or degradation of invasive elements.

### HicA-encoding genes are often located in Defense Islands

Prokaryotic Defense Islands are regions enriched in antivirus defense systems and mobile genetic elements (MGEs), including prophages, integrative and conjugative elements, transposons, recombinases, and IS sequences (2, 3). Except for the CRISPR-Cas systems, different classes of defense systems, including TA and restriction-modification systems, exhibit clustering in Defense Islands. To further strengthen the argument that HicA-domains function in phage defense, we engaged in identifying HicA-domains encoded by Defense Islands. The newly developed online tool TADB3.0 facilitates the identification of TA loci encoded by MGEs. As an example, TADB3.0 has cataloged 93 strains of *Shigella flexneri*, among which 40 harbor *hicAB* or *hicBA* loci within MGEs (52). A comparison of Defense Islands from different *S. flexneri* strains encoding *hicAB* is shown in Fig. S15A. Thus, TAs encoding HicA domains are often located within Defense Islands.

### TA genes often cluster

During the manual inspection of DNA sequences encoding *hicA* and *hicB* genes, it often became apparent that other TA loci were encoded by neighboring regions. In the archaeon *Methanosarcina barkeri* 3, a Defense Island encodes two *hicBA* loci, two solitary *hicB* genes and one *vapBC* locus (Fig. S15B). In a *Thermodesulfobacterium hydrogeniphilum* strain, a Defense Island encodes three *hicBA*, three *relBE*, two *vapBC,* and a solitary *vapC* gene, in total eight different TA loci in a region of 7 kilobases of DNA. Furthermore, TA loci often reside in close proximity to transposases or IS elements (52), consistent with their high lateral mobility in Defense Islands.

### Phylogeny of HicA toxins

**Fig 7:**
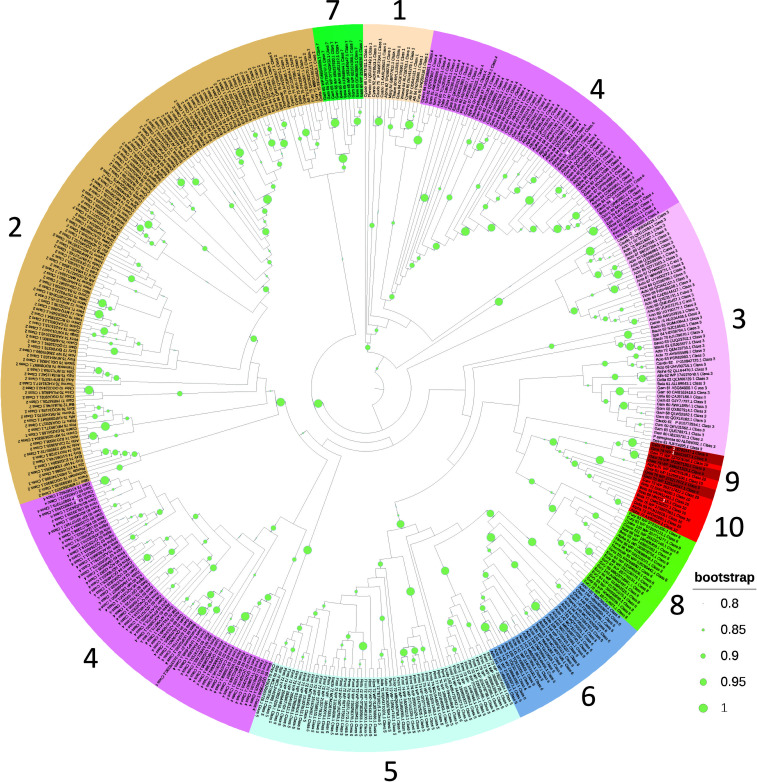
Phylogenetic tree of Classes 1 to 10 HicAs. HicA sequences were aligned by Kalign, tree reconstruction was performed by the FastTree module of Geneious Prime and visualized by iTOL. Numbers refer to Classes 1 to 0. The tree was pruned for outliers.

The phylogenetic analysis of HicA sequences, spanning Classes 1 to 10, yields insights into the evolutionary relationships among the toxins. Significantly, members of each class tend to form clusters, reflecting their shared structural and functional characteristics, except for Class 4, which bifurcates into two distinct clusters (Fig. 7). Classes 1 and 2 appear to evolve independently, hinting at separate evolutionary trajectories, despite their similar sizes and the absence of a DNA-binding domain. Similarly, Classes 7 and 8 constitute entirely distinct branches on the tree, consistent with their distinct HicBs. Conversely, the HicAs of Classes 5 and 6, as well as Classes 9 and 10, exhibit a clear pattern of clustering with robust statistical support, suggesting a close genetic relationship between these pairs. However, it is important to note that due to the relatively small size and high sequence variability of HicA domains, the statistical significance of certain branches in the phylogenetic tree may be somewhat limited.

### Conclusion

The comprehensive analysis of HicA toxin domains has uncovered a diverse array of genetic organizations and functional associations. This study not only identified Classes 3 and 4 but also introduced 12 new gene classes encoding HicA toxin domains, significantly expanding our understanding of these systems. Several noteworthy findings and questions have emerged from this investigation: Classes 1 and 2, being the most prevalent, raise the question about the regulation of their gene expression, particularly considering the absence of an identifiable DNA-binding domain in HicB; the reversal in gene order between Classes 3 and 4; the reversal in gene order and transition from HTH/RHH DBD to Phd/YefM DBD in Classes 5 and 6 highlights the variations in DNA-binding modes associated with these gene classes; the identification of the Shared Domain in Class 6 HicA and some RelEs suggests potential functional connections between *hicBA* and *relBE* systems, consistent with a common role in phage-defense; the proximity of Class 9 and 10 HicA-encoding genes to pVip-encoding genes, as well as the occasional association of RelE-encoding genes with pVip genes, also suggests potential cooperation in anti-phage defense; classes 11 to 13 HicA domains are encoded by fused *hicAB* or *hicBA* monocistronic operons, resembling the anti-phage gene CapRelSJ46 and these arrangements are also consistent with a mechanism for curbing phage infections; Class 14, featuring an SMC domain fused to a HicA domain, might also play a role in defense against phages, plasmids, or other mobile genetic elements, akin to the Wadjet and Lamassu systems although alternative functions cannot be ruled out. In conclusion, this study provides substantial support for the proposition that genes encoding HicA domains play pivotal roles in defense against phages, plasmids, and other mobile genetic elements.

## MATERIALS AND METHODS

### Data sampling

Sequences of experimentally validated HicA toxins were used as seeds in BLASTP searches at NCBI (https://blast.ncbi.nlm.nih.gov/), using different bacterial phyla as search spaces (Table S1). HMMSEARCH at ebi.ac.uk (53) was used to expand poorly populated Clades. In Table S1, kept HicA sequences are less than 95% identical to any other kept HicA sequence.

### TA gene organization and gene neighborhood analysis

were accomplished using webFlaGs (54) or TADB3.0 (52). The gene organizations shown in Fig. 2 were then confirmed by manual inspection of the DNA regions encoding the HicAs of Table S1.

### Sequence alignments and phylogenetic trees

Sequence alignments were generated by Clustal Omega (55) or Kalign (56) at www.ebi.ac.uk and imported into Jalview (57). Protein sequence alignments in Jalview 2.11.0 were exported as vector files (EPS or SVG formats), converted to jpg files, and imported into Adobe Illustrator, annotated and saved in PDF format for publication. Phylogenetic trees were visualized using iTOL (58). Reconstruction of phylogenetic trees was accomplished using FastTree (59) via the CIPRES module in Genious Prime which uses the Maximum Likelihood approach and Ultrafast bootstrapping.

### Protein structure prediction, protein similarity searches, and protein structure visualization

Protein secondary structures were predicted from sequence alignments using the link to JPred (60) in JalView. Protein tertiary structures were modeled using AlphaFold2 (61) via the ColabFold v1.5.2-patch (62) or the AlphaFold patch of ChimeraX. Mutimeric structures were modeled by MultiFOLD (63) or AlphaFold2 and validated by ModFOLDdock (64). Structure similarity searches were done using Phyre2 (65) or FoldSeek (66). Structures were visualized and annotated using ChimeraX (Pettersen et al., 2021). HTH motifs were identified by EMBOSS (67).

### Categorization of HicB antitoxins encoded by Class 1 *hicAB* operons

Class 1 *hicB* genes encode HicB lacking a DBD. These *hicB* genes could encode either a functional protein or a pseudogene that had arisen by mutation, e.g., a stop-codon mutation that truncated the *hicB* gene. To analyze if this was the case, each individual Class 1 HicB protein sequence was used as a query in BLASTP searches at NCBI (https://blast.ncbi.nlm.nih.gov/). Some HicBs were almost identical to a HicB with a DBD; operons of this type were discarded. However, most HicB antitoxins exhibited a BLASTP search pattern very similar to other HicBs, revealing homologs of similar sizes but with more distantly related protein sequences, indicating that these proteins were actively evolving even though they lacked a DBD. These proteins were included in the Class 1 *hicBA* operons. Furthermore, the relative uniform size distribution of Class 1 HicBs (60 to 87 aa; Table S1) is consistent with the notion that the antitoxins are not products of randomly mutated *hicB* genes.

## SUPPLEMENTAL MATERIAL

Supplemental Material mbio.03293-23-s0001.pdf Supplemental figures and table legend.

Table S1 mbio.03293-23-s0002.xlsx Genetic contexts of HicA domain-encoding genes.

ASM does not own the copyrights to Supplemental Material that may be linked to, or accessed through, an article. The authors have granted ASM a non-exclusive, world-wide license to publish the Supplemental Material files. Please contact the corresponding author directly for reuse.
