# Supplementary material for: Diverse genetic contexts of HicA toxin domains propose a role in anti-phage defense
Source: mBio. 2024 Jan 18;15(2):e03293-23. doi: 10.1128/mbio.03293-23 (PMC10865869; doi:10.1128/mbio.03293-23)
Supplement: Supplemental Material — Supplemental figures and table legend. [file mbio.03293-23-s0001.pdf]

Supplemental Material

Diverse Genetic Contexts of HicA Toxin Domains Propose a Role in Anti-Phage Defense

Kenn Gerdes

Legends of Supplementary Figures

Figure S1. HicB of *E. coli* K-12 exhibits a partial RNase H fold.

(**A**) Comparison of experimental secondary structures of HicB of *E. coli* K-12 (P67697.2, upper) and *T. thermophilus* HB8 (BAD71735.1, lower).

(**B**) Tertiary structures of the N-terminal partial RNase H-like domain of HicB of *E. coli* K-12 (6HPB.pdb, left), TTHA1013 of *T. thermophilus* HB8 (1WV8.pdb, middle) and their superimposition (right) with an RMSD between 18 pruned atom pairs of 1.4Å. These structures were obtain experimentally (1, 2).

Figure S2. Sequence alignments of Class 5 and 6 HicA.

(**A**) Alignment of Class 6 HicAs. The Shared Domain is indicated with a bar.

(**B**) Alignment of Class 5 and 6 HicAs. Class 5 HicAs are typical small, mono-domain proteins with a dsRBD (**Table S1**) while Class 6 HicAs are two domain proteins consisting of a typical N-terminal dsRBD and a second domain here called the Shared Domain of 50 to 55 amino acids. Conserved proline residues (green) may serve as domain-breakers between the two domains.

Figure S3. Sequence alignments of RelE/ParE toxins with and without the Shared Domain.

(**A**) Alignment of RelE/ParE superfamily of toxins with the typical N-terminal RNase I domain and a Shared C-terminal Domain (70 to75 aa) .

(**B**) Alignment of typical mono-domain RelE/ParE toxins and two-domain RelE/ParE toxins with a Shared C-terminal Domain.

Figure S4. Sequence alignments of the Shared Domains of Class 6 HicAs and RelE/ParE.

(**A**) Alignment of the Shared Domains of Class 6 HicAs.

(**B**) Alignment of Shared domains of RelE/ParE family toxins.

(**C**) Alignment of the two classes of Shared Domains.

Figure S5. Comparison of Quaternary Structure Models of $RelB_2RelE_2$ Complexes.

(**A**) Experimental $RelB_2E_2$ tetramer model of canonical RelE (CAA26251.1) and RelB (CAA26250.1) of *E. coli* K-12 (3).

(**B**) $RelB_2E_2$ tetramer model in which RelE has a Shared Domain (WP_141355914.1) and RelB (WP_174787664.1) of *Glutamicibacter nicotianae* (an Actinomycete) generated by MultiFOLD (pLLDT = 0.67). The Shared Domains are marked with green circles. Note that RelB in (A) dimerizes via a HTH domain while RelB in (B) dimerizes via a Phd/YefM Domain.

Figure S6. Tertiary and Quaternary Structure Models of Class 7 HicA, HicB and HicAB.

(**A**) Predicted model of Class 7 HicA (WP_001674386.1; 65 aa) revealing the canonical dsRBD α-β-β-β-α fold of HicA.

(**B**) Predicted Model of Class 7 HicB (WP_000065196.1; 217 aa) revealing its two-domain structure.

(**C**) Dimer structure of Class 7 HicA and HicB modelled by MultiFold. Dimer plDDT: 0.918; Dimer pTM: 0.750; Assemby quality: 0.9098; Interface quality: 0.9058.

Figure S7. Secondary and Tertiary Structures of Class 8 HicA and HicB.

(**A**) Tertiary structure of Class 8 HicA (GenBank ID: WP_004069339.1; 95 aa) modelled by AF2.

(**B**) Tertiary structure of Class 8 HicB (GenBank ID: WP_004069337.1; 342 aa) modelled by AF2. See the text and **Table S1** for further details.

(**C**) Sequence alignment and secondary structure prediction of Class 8 HicA sequences. The prediction was generated by analysis of an alignment of Class 8 HicA sequences in the JPRED module of Jalview. α-helices are colored magenta and β-sheets cyan.

Figure S8. Sequence Alignments and Secondary Structures of Class 8 HicB sequences.

Sequence alignment and secondary structure prediction of Class 8 HicB sequences. The prediction was generated by analysis of an alignment of Class 8 HicB sequences in the JPRED module of Jalview. α-helices are colored red and β-sheets green.

Figure S9. Tertiary Structure Models of Class 10 HicAs.

The tertiary structures of 21 Class 10 HicAs were modelled be AlphaFold2. They all have a pLLDT score > 90. Numbers refer to the listing in **Table S1** (Sheet #19).

Figure S10. RelE-Encoding Genes Adjacent to pVip Genes.

(**A**) Comparison of the organization of genes encoding pVip and HicA, and pVip and RelE.

(**B**) Superposition of RelE from a pVip-encoding operon (WP_263530310.1; cyan) and RelE-homolog YoeB of *E. coli* K-12 determined experimentally (4) (2A6S; magenta). The RMSD between 19 pruned atom pairs is 0.835 Å.

(**C**) Sequence alignment and secondary structure prediction of 15 RelE-homologous RNases encoded by genes juxtaposed to pVip-encoding genes and RelE of *E. coli* K-12 (bottom).

Figure S11. Class 11 Fused HicAB Dimer Model Structures.

(**A**) Predicted dimer model of Class 11 fused HicAB (KPW96986.1). The monomers dimerizes via their RHH domains that are superimposed on a plasmid-encoded CopG dimer shown in blue (5) (RMSD between 25 pruned atom pairs is 0.753Å).

(**B**) Predicted dimer model of fused Class 11 HicBA (WP_243550782.1). The monomers dimerizes via their HTH domains that are superimposed on the HTH domain of HipB of *E. coli* K-12 (6) (RMSD between 35 pruned atom pairs is 1.226Å).

Figure S12. Phylogenetic Tree of 185 Class 2 HicBAs generated by fusion in silico of the HicB and HicA proteins and 7 Class 12 naturally fused HicBAs. The HicBA sequences were aligned with Clustal Omega and the Tree generated by the FastTree module of Geneious Prime using Ultrafast bootstrapping. Blue: Class 2 HicBA sequences; Red: Class 12 HicBA sequences

Figure S13. Secondary and Tertiary Structures of a Fused Class 13 HicBA Toxin – Antitoxin.

(**A**) Secondary structure of Class 13 HicBA (WP_067334119.1). Helices are in magenta, sheets in cyan and coil in light grey.

(**B**) Tertiary structure of Class 13 HicBA (WP_067334119.1). Helices are in magenta, sheets in cyan and coil in light grey.

Figure S14. Predicted Secondary and Tertiary Structures of a Fused SMC-HipA Protein.

(**A**) Primary and secondary structures of a Class 14 SMC-HicA two-domain protein (WP_223300692.1). Helices are in magenta, sheets in cyan.

(**B**) Tertiary structure of Class 14 SMC-HicA protein (WP_223300692.1). The coloring palette was from AlphaFold2 where blue is high and yellow low quality of the prediction. In particular, loops and parts of the coiled-coil domains were predicted with low quality whereas the C-terminal HicA domain and a globular domain were predicted with high quality.

(**C**) Enlargement of the C-terminal HicA-domain of the structure shown in (B).

Figure S15. Defense Islands Encoding *hicAB* and *hicBA* Genes.

(**A**) Alignment of DNA sequences encoding Defense Islands of 11 *Shigella flexneri* strains encoding *hicAB* loci. The *hicA* and *hicB* genes are colored red and green, respectively, transposase genes are yellow and virulence factors magenta. The data were retrieved from TADB 3.0 and the Figure generated by Clinker & Clustermap (7) embedded in TADB3.0. (8).

(**B**) Defense Island of archaeon *Methanosarcina barkeri* 3 (NZ_CP009517.1) encoding two *hicBA* green, red), two solitary *hicB* genes (green) and a *vapBC* locus (pale blue, blue).

(**C**) Defense Island of *Thermodesulfobacterium hydrogeniphilum* strain DSM 14290 (NZ_JQKW01000008) encoding three *hicBA* (green, red), three *relBE* (magenta, pink), two *vapBC* (pale blue, blue) and one *vapC* gene (blue). TPR: gene encoding a tetratricopeptide repeat protein. Genes unrelated to TA modules are shown in grey. DUF: domain of unknown function. DUF2442 is often found in antitoxins (9).

Supplementary Table

Table S1: Primary information of members of the 14 Classes of HicA-domain encoding TA modules identified in this work.

Sheets 1 to 14: Information of HicA and HicB of the 14 classes. Columns A to J yield the following information: (**A**) GenBank ID of HicA-domain protein; (**B**) Organism; (**C**) Label yielding phylum, gene length in codons, GenBank ID and Class of HicA-domain protein. These labels were used in sequence alignments and in the phylogenetic trees (**Figures 7 and S12**); (**D**) HicA sequence; (**E**) Distance between *hicA* gene and its cognate antitoxin-encoding gene in nucleotides (a minus indicates gene overlap); (**F**) Genetic order (GO) of each toxin – antitoxin module; (**G**) HicA Class; (**H**) Label yielding HicB antitoxin phylum, gene length, GenBank ID and Class; (**I**) HicB Antitoxin or pVIP sequence; (**J**) Domains in HicB and comments to each individual entry in the Table.

Sheet 15: *relBE* / *parDE* modules where the toxins contain the C-terminal Shared Domain also present in Class 6 HicA.

Sheet 16: Anti-phage modules encoding a pVip and an adjacent RelE toxin.

Sheet 17: List of Phyla abbreviations used in Sheets 1 to 16.

Supplementary References

1. Hattori M, Mizohata E, Manzoku M, Bessho Y, Murayama K, Terada T, Kuramitsu S, Shirouzu M, Yokoyama S. 2005. Crystal structure of the hypothetical protein TTHA1013 from Thermus thermophilus HB8. Proteins 61:1117-20.
2. Manav MC, Turnbull KJ, Jurenas D, Garcia-Pino A, Gerdes K, Brodersen DE. 2019. The E. coli HicB Antitoxin Contains a Structurally Stable Helix-Turn-Helix DNA Binding Domain. Structure 27:1675-1685 e3.
3. Bøggild A, Sofos N, Andersen KR, Feddersen A, Easter AD, Passmore LA, Brodersen DE. 2012. The crystal structure of the intact E. coli RelBE toxin-antitoxin complex provides the structural basis for conditional cooperativity. Structure 20:1641-8.
4. Kamada K, Hanaoka F. 2005. Conformational change in the catalytic site of the ribonuclease YoeB toxin by YefM antitoxin. Mol Cell 19:497-509.
5. Gomis-Ruth FX, Sola M, Acebo P, Parraga A, Guasch A, Eritja R, Gonzalez A, Espinosa M, del Solar G, Coll M. 1998. The structure of plasmid-encoded transcriptional repressor CopG unliganded and bound to its operator. EMBO J 17:7404-15.
6. Schumacher MA, Balani P, Min J, Chinnam NB, Hansen S, Vulic M, Lewis K, Brennan RG. 2015. HipBA-promoter structures reveal the basis of heritable multidrug tolerance. Nature 524:59-64.
7. Gilchrist CLM, Chooi YH. 2021. clinker & clustermap.js: automatic generation of gene cluster comparison figures. Bioinformatics 37:2473-2475.
8. Guan J, Chen Y, Goh YX, Wang M, Tai C, Deng Z, Song J, Ou HY. 2023. TADB 3.0: an updated database of bacterial toxin-antitoxin loci and associated mobile genetic elements. Nucleic Acids Res doi:10.1093/nar/gkad962.
9. Makarova KS, Wolf YI, Koonin EV. 2009. Comprehensive comparative-genomic analysis of type 2 toxin-antitoxin systems and related mobile stress response systems in prokaryotes. Biol Direct 4:19.

Figure S1

A

	1 ... 10 ... 20 ... 30 ... 40 ... 50 ... 60 ... 70
E. coli K-12 HicB	MRYPVTLTPAPEGGYMVSFVDIPEALTQGETVAEAMEAAKDALLTAFDFYFEDNELIPLPSPLNSHDHFIEV
T. thermo. TTHA103	MRTLKVQALWDGEAGVWVAESDDVPGLATEAATLEELLAKLAVMVPELLEENGVALELPVELRLEATRPLVFS

B

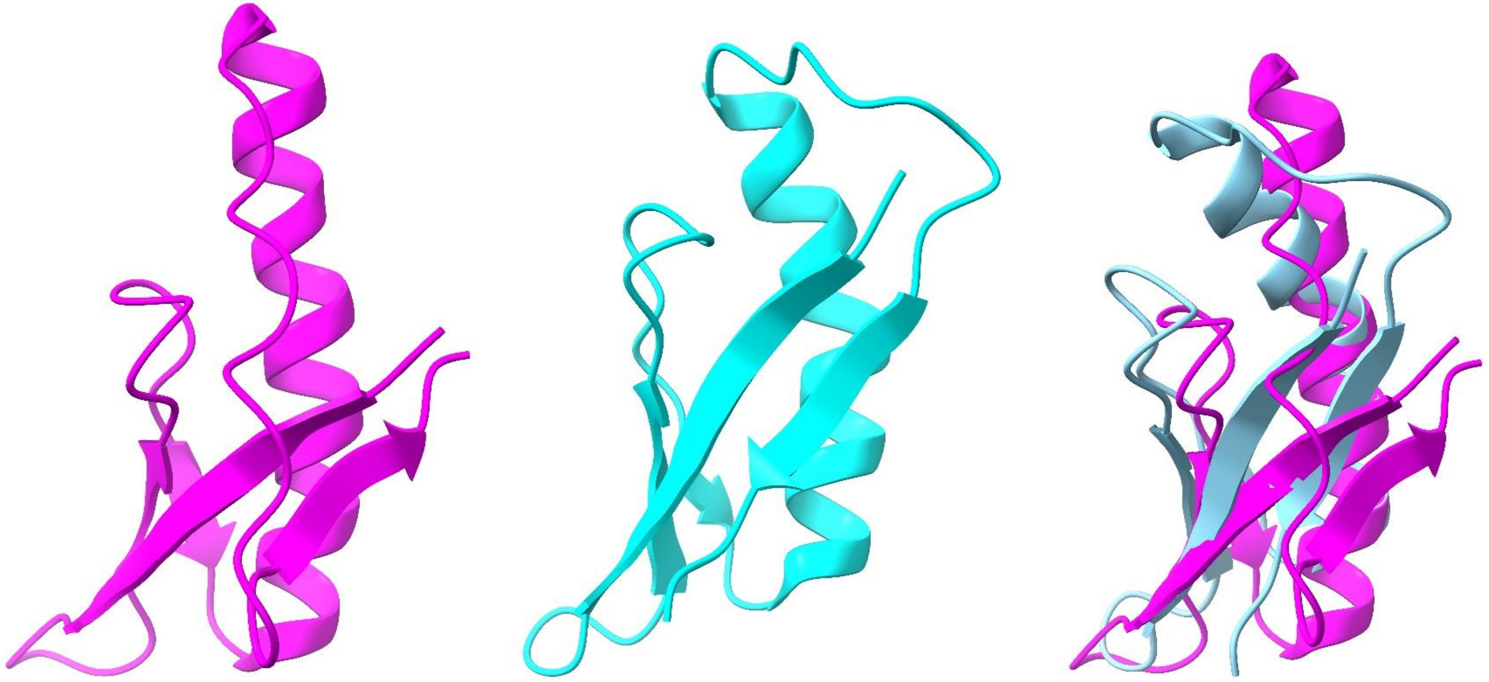

Figure S2

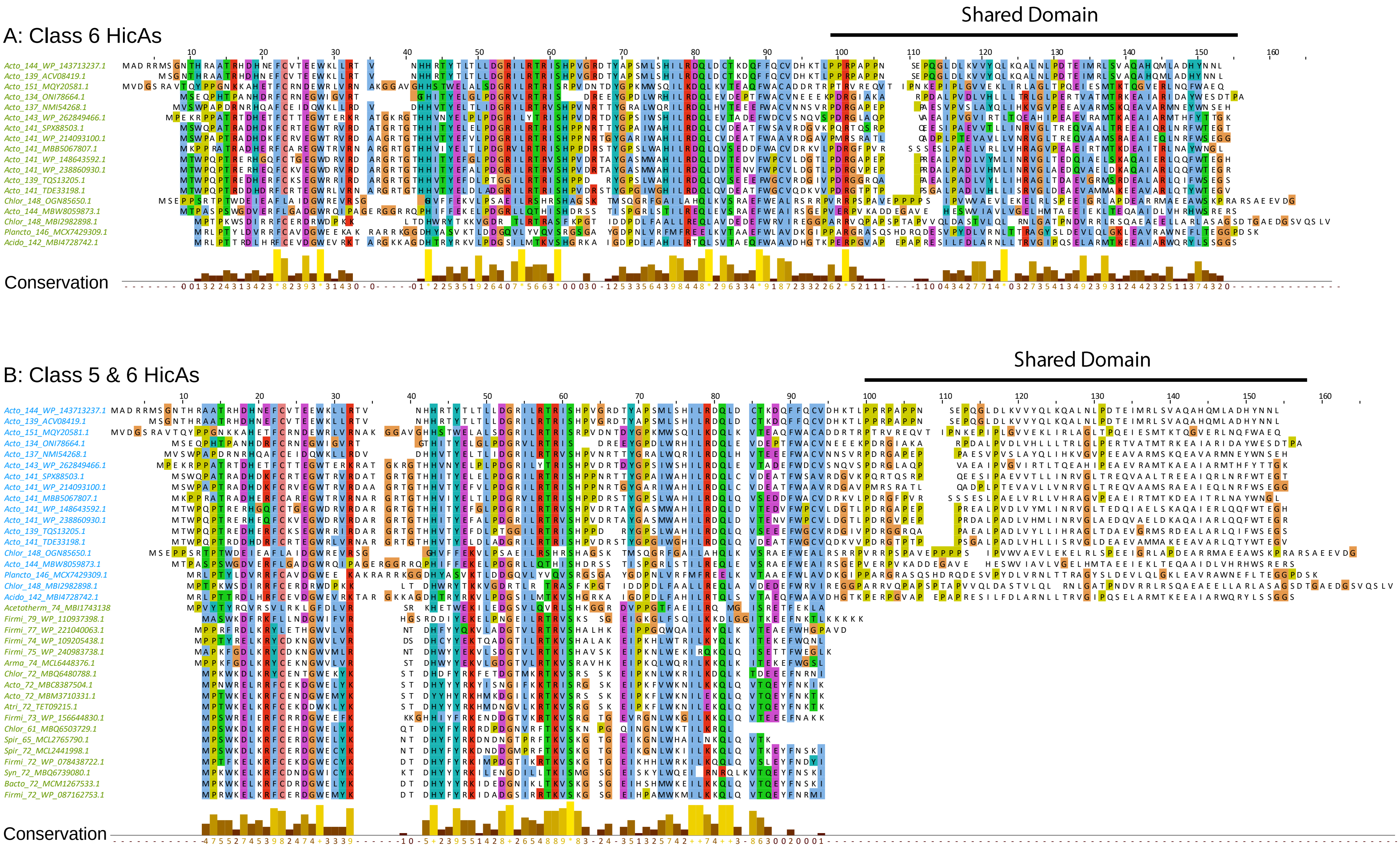
A: Class 6 HicAs
Shared Domain
Acto_144_WP_143713237.1
Acto_139_ACV08419.1
Acto_151_MQY20581.1
Acto_134_ONI78664.1
Acto_137_NMI54268.1
Acto_143_WP_262849466.1
Acto_141_SPX88503.1
Acto_141_WP_214093100.1
Acto_141_MBB5067807.1
Acto_141_WP_148643592.1
Acto_141_WP_238860930.1
Acto_139_TQS13205.1
Acto_141_TDE33198.1
Chlor_148_OGN85650.1
Acto_144_MBW8059873.1
Chlor_148_MBI2982898.1
Plancto_146_MCX7429309.1
Acido_142_MBI4728742.1
Conservation
B: Class 5 & 6 HicAs
Shared Domain
Acetotherm_74_MBI1743138
Firmi_79_WP_110937398.1
Firmi_77_WP_221040063.1
Firmi_74_WP_109205438.1
Firmi_75_WP_240983738.1
Arma_74_MCL6448376.1
Chlor_72_MBQ6480788.1
Acto_72_MBC8387504.1
Acto_72_MBM3710331.1
Atri_72_TET09215.1
Firmi_73_WP_156644830.1
Chlor_61_MBQ6503729.1
Spir_65_MCL2765790.1
Spir_72_MCL2441998.1
Firmi_72_WP_078438722.1
Syn_72_MBQ6739080.1
Bacto_72_MCM1267533.1
Firmi_72_WP_087162753.1
Conservation

Figure S3

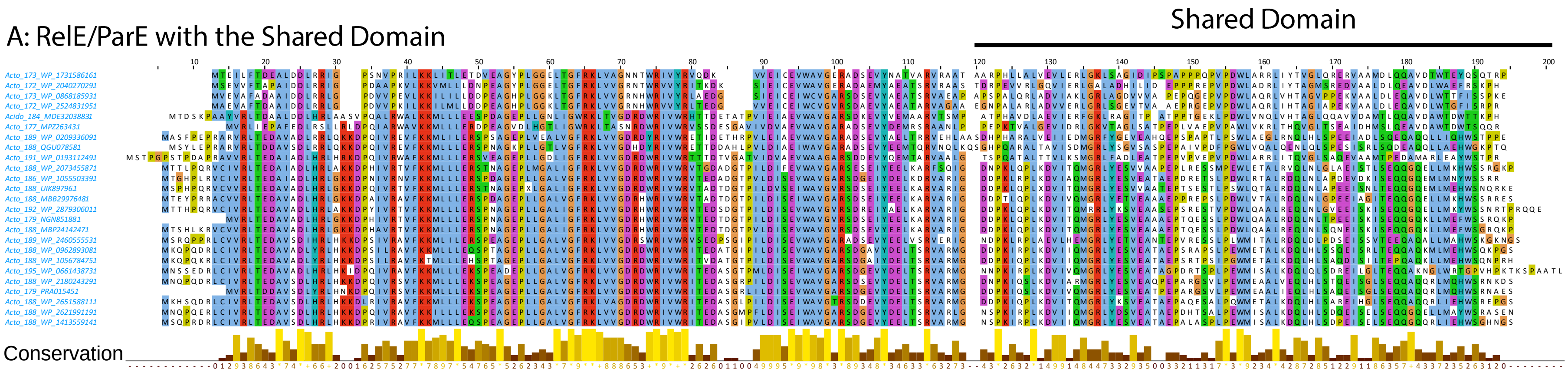
A: RelE/ParE with the Shared Domain
Shared Domain
Conservation

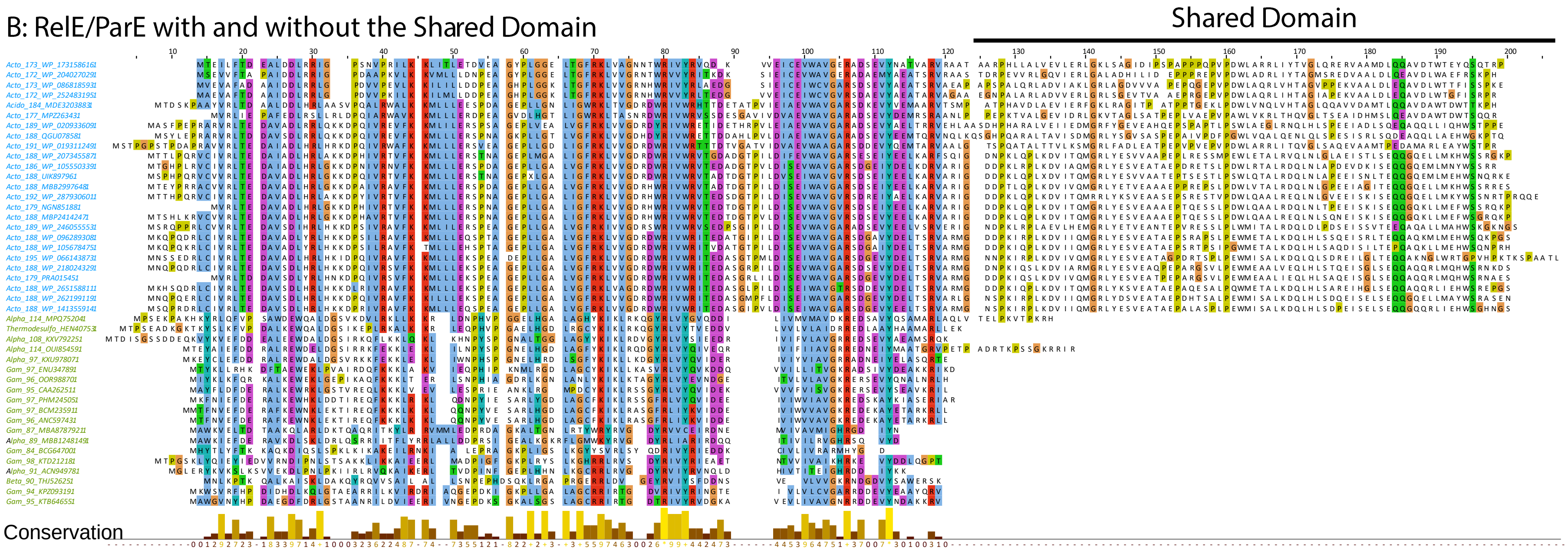
B: RelE/ParE with and without the Shared Domain
Shared Domain
Conservation

Figure S4

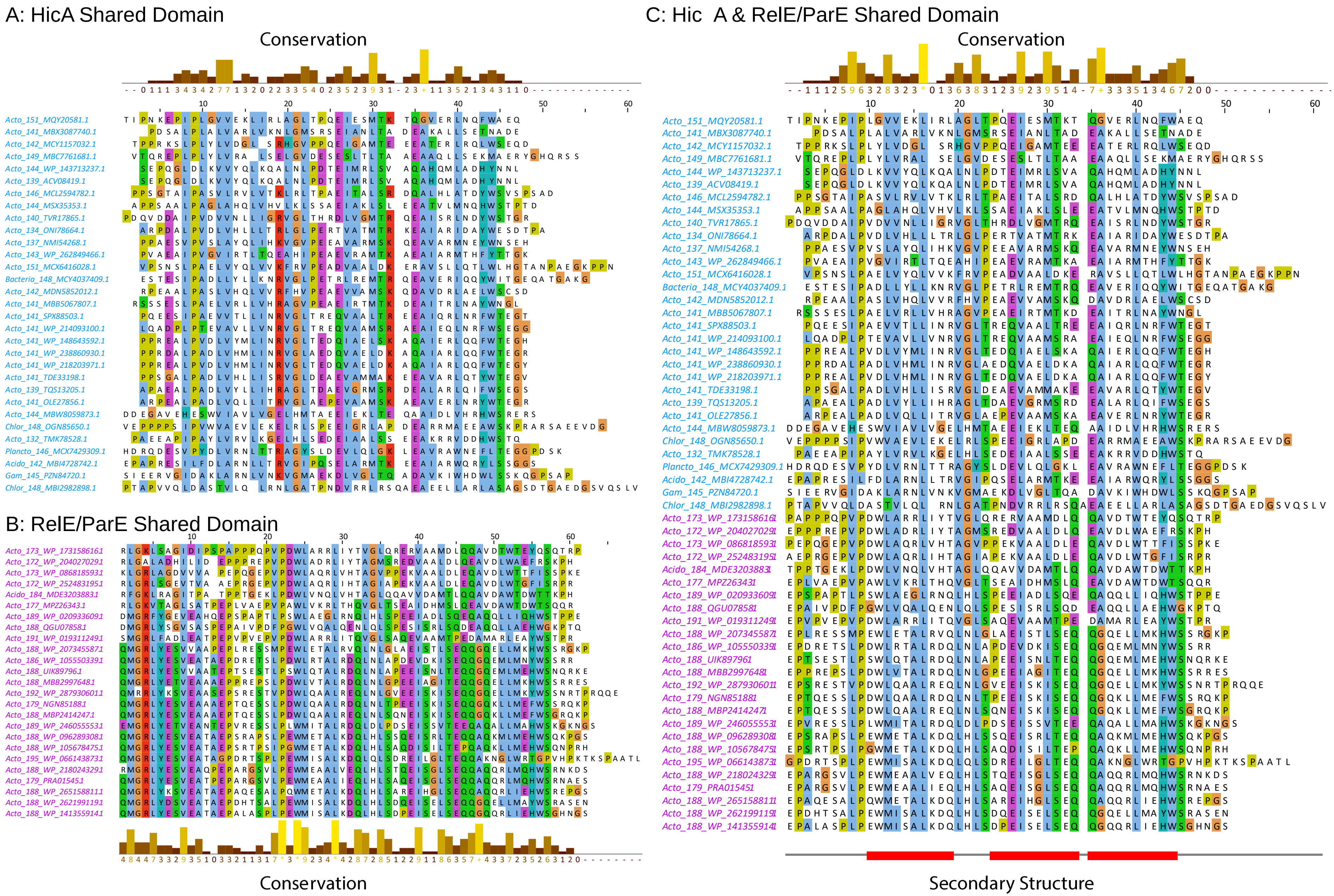

A: HicA Shared Domain
Conservation
B: RelE/ParE Shared Domain
Conservation
C: Hic A & RelE/ParE Shared Domain
Conservation
Secondary Structure

Figure S5

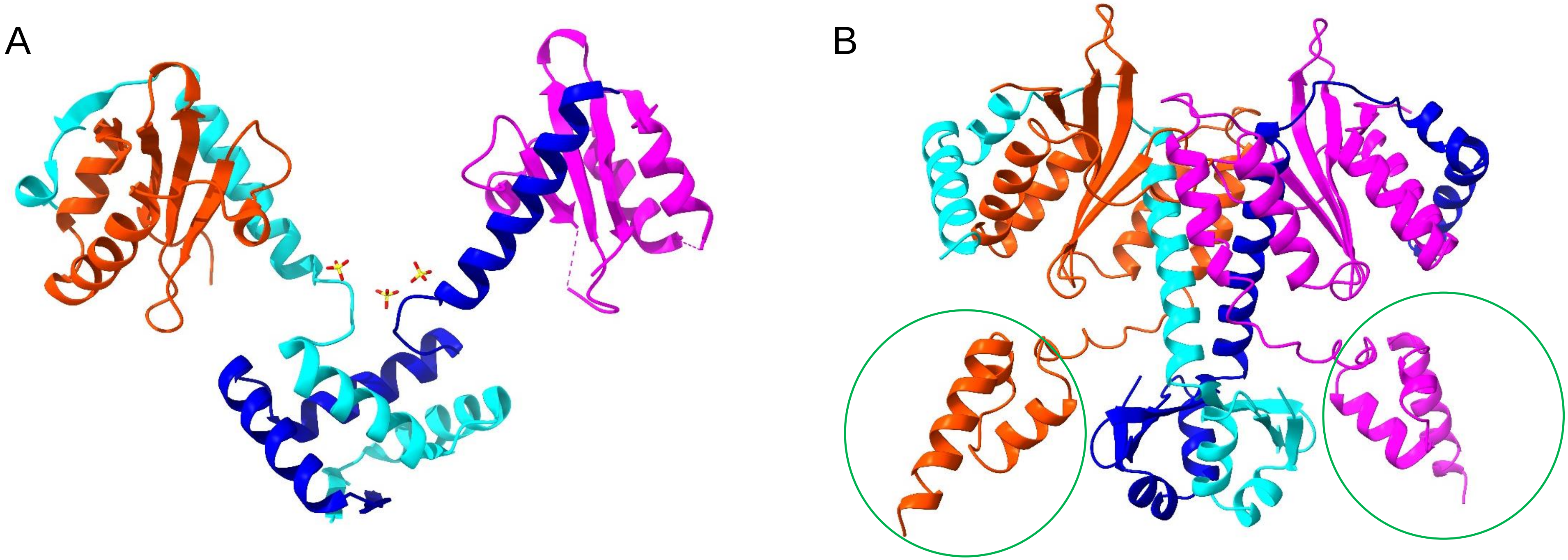

Figure S6

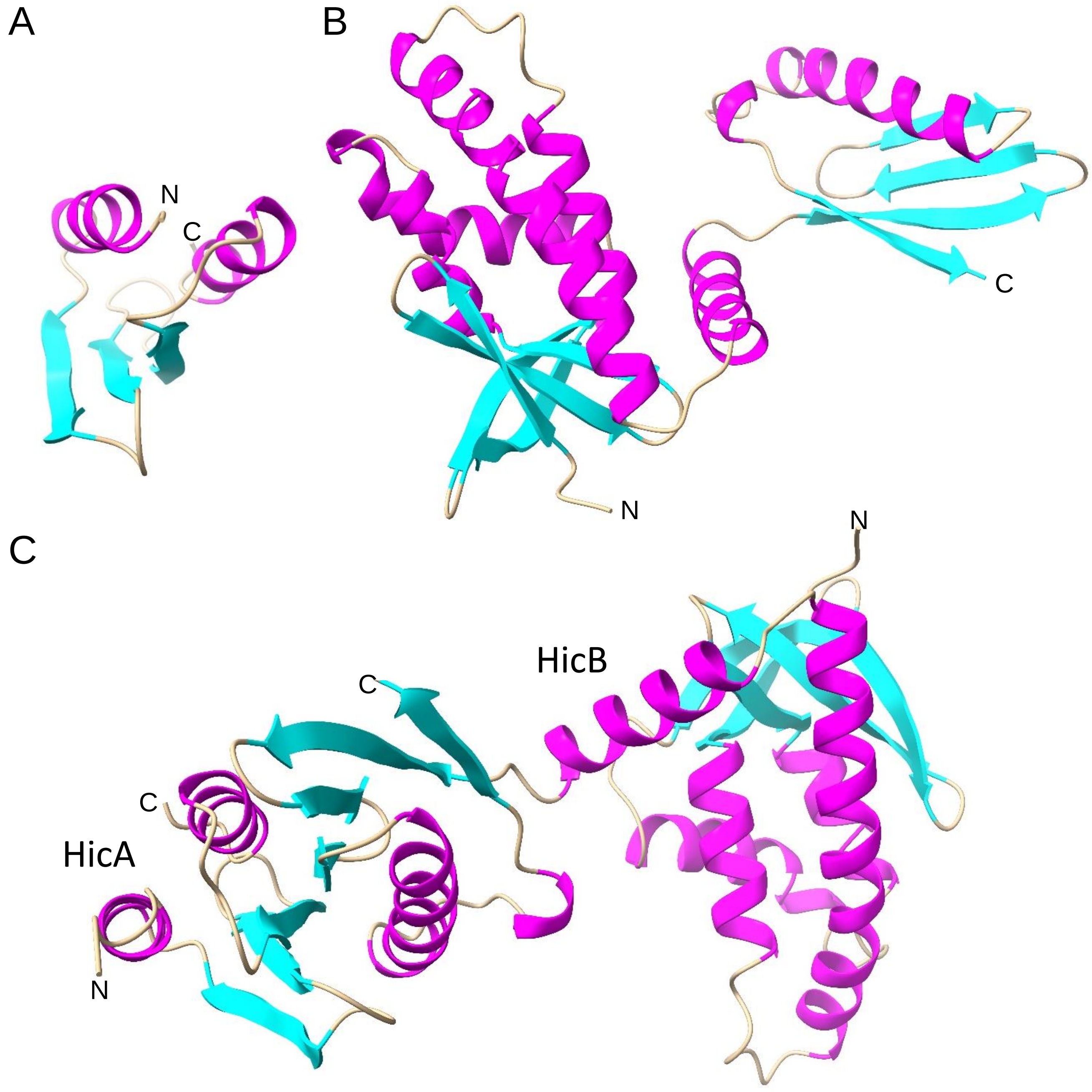

Figure S7

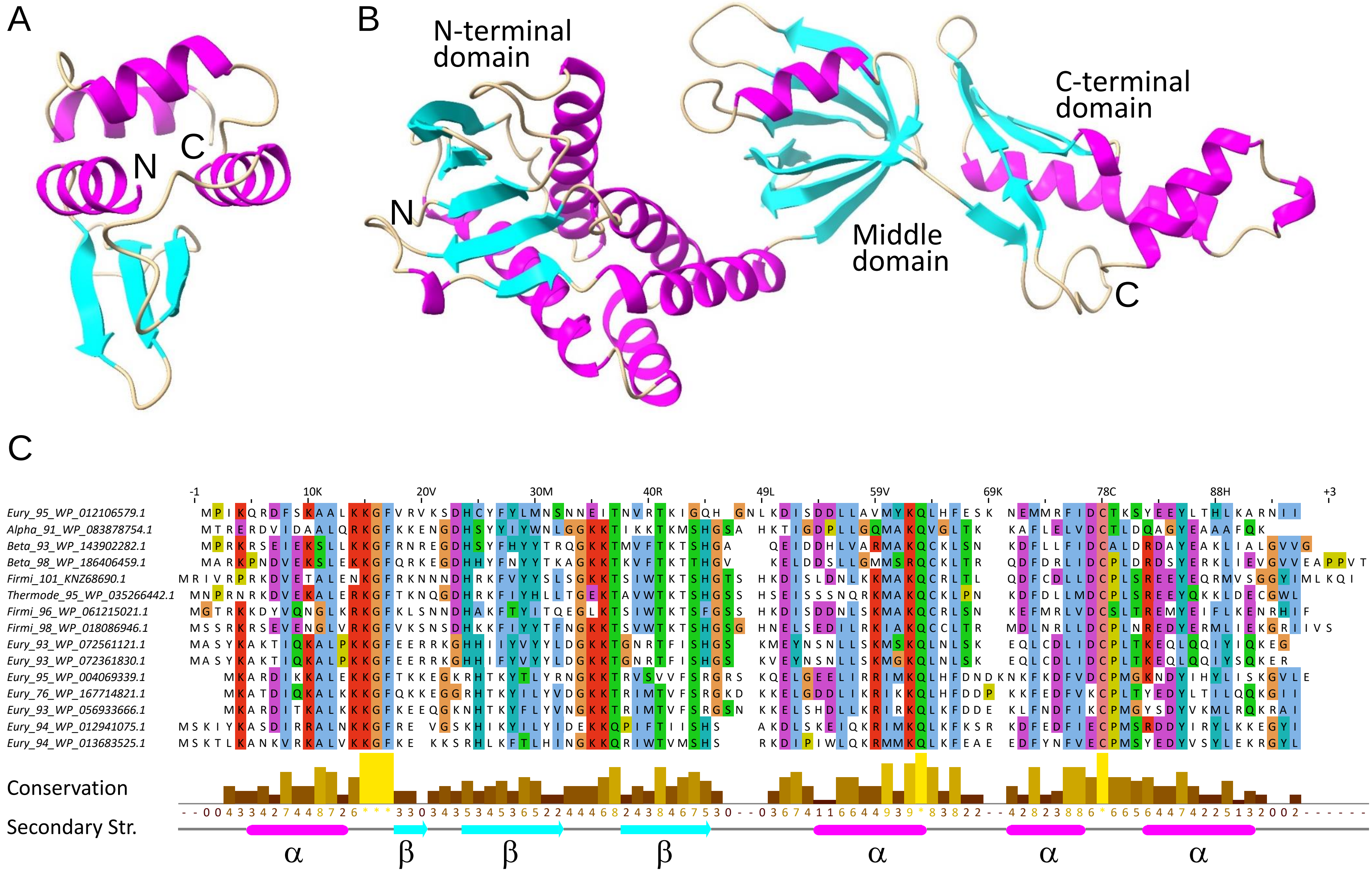

A
N
C
B
N-terminal domain
Middle domain
C-terminal domain
N
C
C
Eury_95_WP_012106579.1
Alpha_91_WP_083878754.1
Beta_93_WP_143902282.1
Beta_98_WP_186406459.1
Firmi_101_KNZ68690.1
Thermode_95_WP_035266442.1
Firmi_96_WP_061215021.1
Firmi_98_WP_018086946.1
Eury_93_WP_072561121.1
Eury_93_WP_072361830.1
Eury_95_WP_004069339.1
Eury_76_WP_167714821.1
Eury_93_WP_056933666.1
Eury_94_WP_012941075.1
Eury_94_WP_013683525.1
-1 10K 20V 30M 40R 49L 59V 69K 78C 88H +3
Conservation
Secondary Str.
α β β β α α α

Figure S8

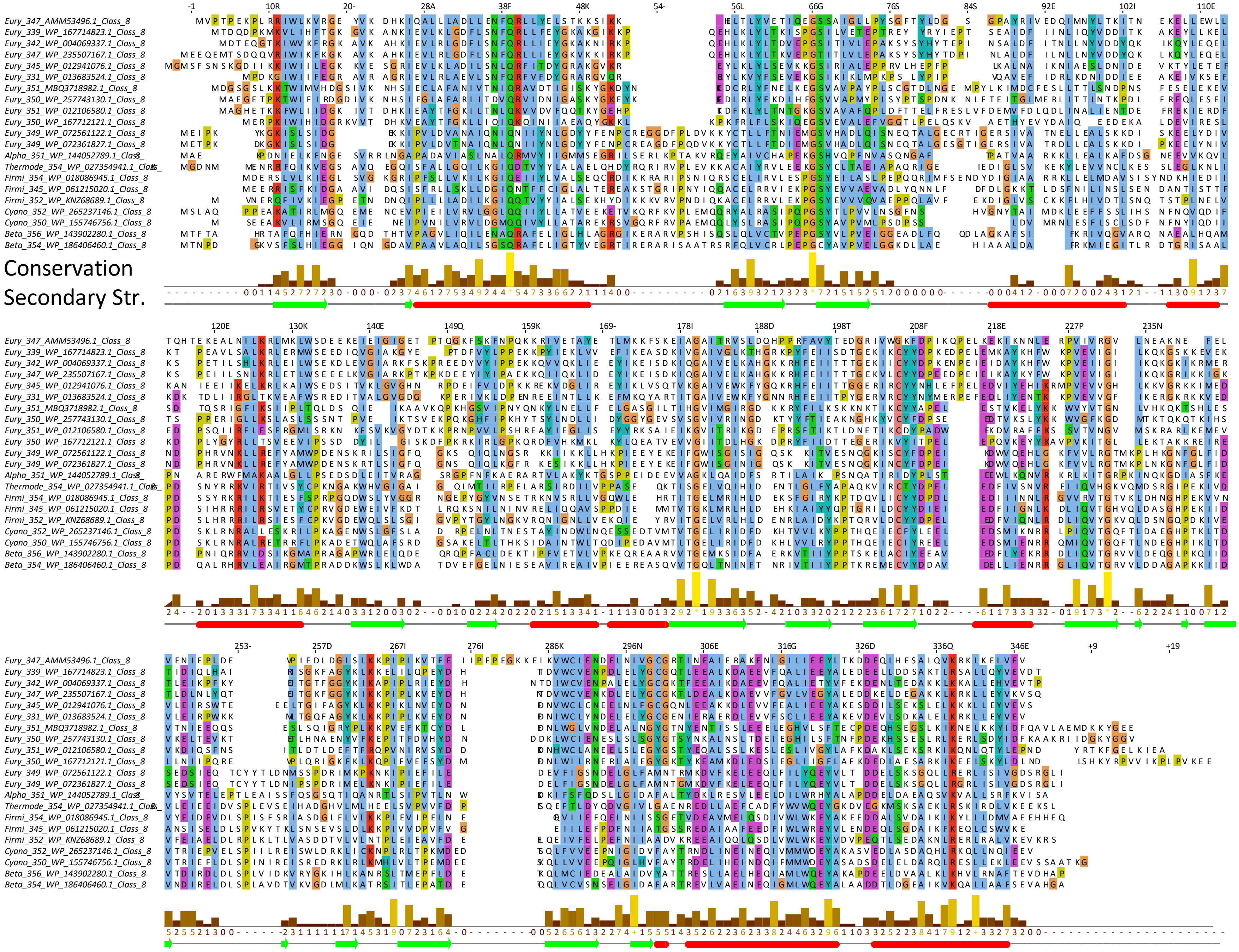
Eury_347_AMM53496.1_Class_8
Eury_339_WP_167714823.1_Class_8
Eury_342_WP_004069337.1_Class_8
Eury_347_WP_235507167.1_Class_8
Eury_345_WP_012941076.1_Class_8
Eury_331_WP_013683524.1_Class_8
Eury_351_MBQ3718982.1_Class_8
Eury_350_WP_257743130.1_Class_8
Eury_351_WP_012106580.1_Class_8
Eury_350_WP_167712121.1_Class_8
Eury_349_WP_072561122.1_Class_8
Eury_349_WP_072361827.1_Class_8
Alpha_351_WP_144052789.1_Class_8
Thermode_354_WP_027354941.1_Class_8
Firmi_354_WP_018086945.1_Class_8
Firmi_345_WP_061215020.1_Class_8
Firmi_352_WP_KNZ68689.1_Class_8
Cyano_352_WP_265237146.1_Class_8
Cyano_350_WP_155746756.1_Class_8
Beta_356_WP_143902280.1_Class_8
Beta_354_WP_186406460.1_Class_8
Conservation
Secondary Str.

Figure S9

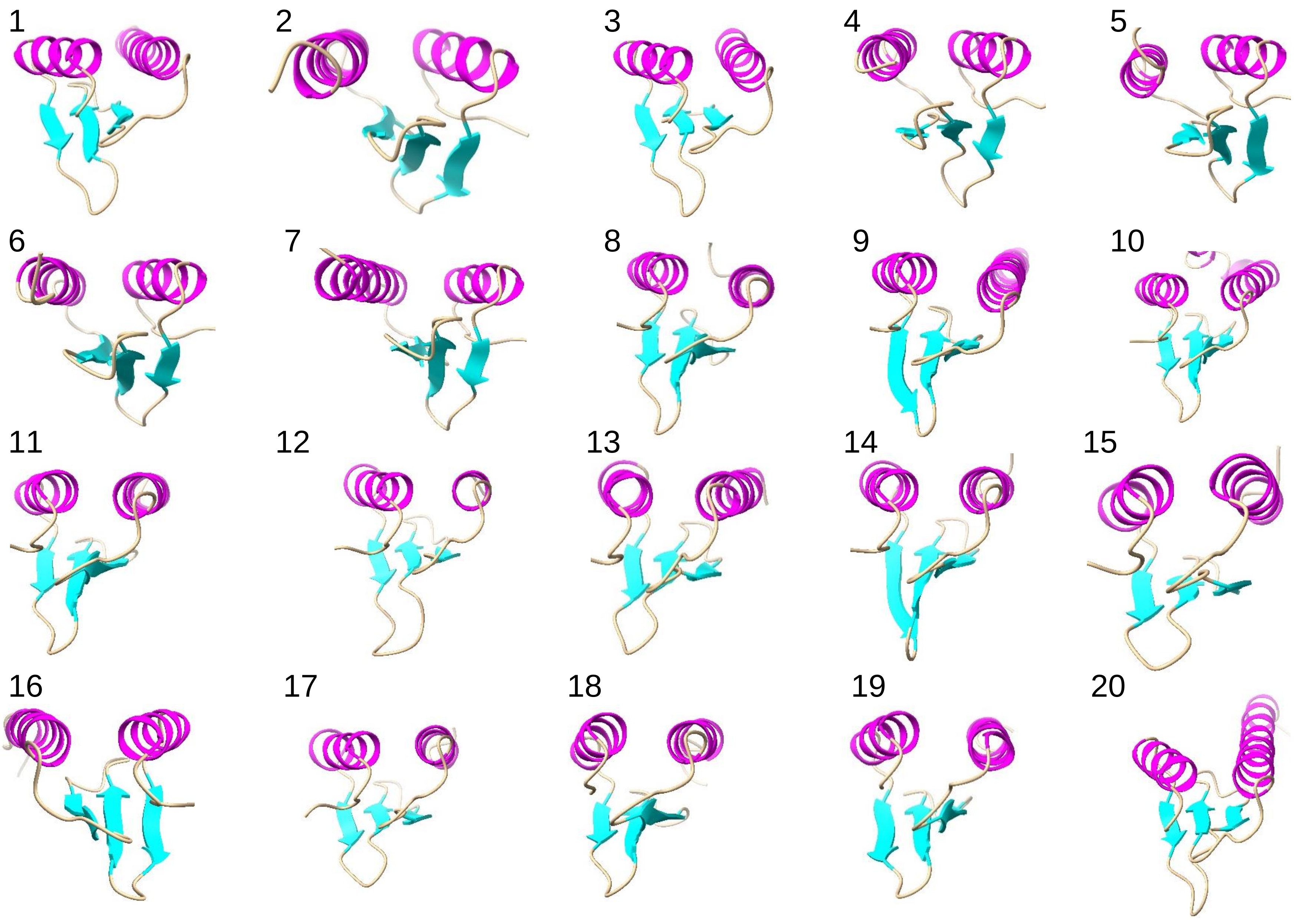

Figure S10

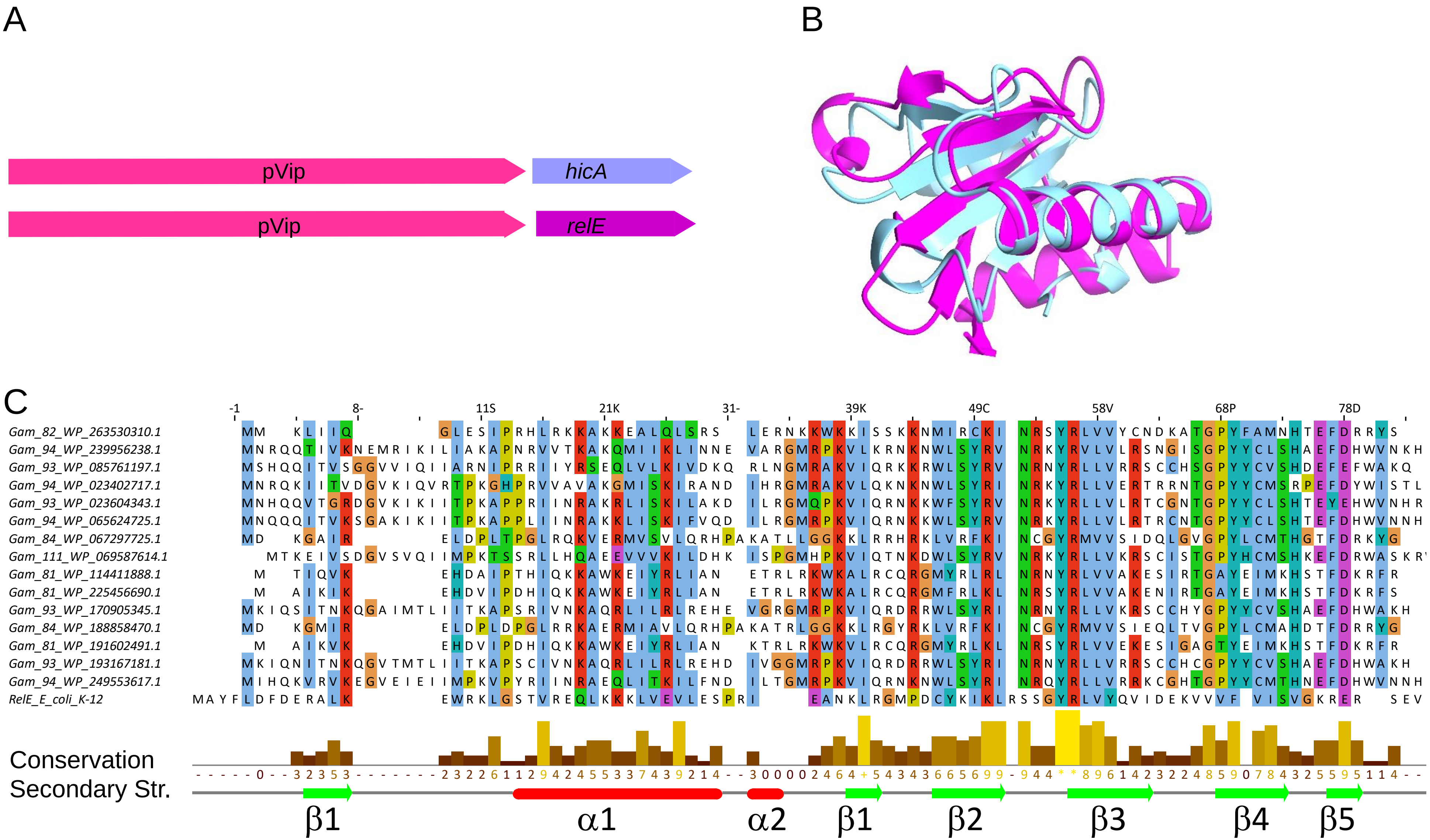

A
pVip
hicA
pVip
relE
B
C
Gam_82_WP_263530310.1
Gam_94_WP_239956238.1
Gam_93_WP_085761197.1
Gam_94_WP_023402717.1
Gam_93_WP_023604343.1
Gam_94_WP_065624725.1
Gam_84_WP_067297725.1
Gam_111_WP_069587614.1
Gam_81_WP_114411888.1
Gam_81_WP_225456690.1
Gam_93_WP_170905345.1
Gam_84_WP_188858470.1
Gam_81_WP_191602491.1
Gam_93_WP_193167181.1
Gam_94_WP_249553617.1
RelE_E_coli_K-12
Conservation
Secondary Str.
β1
α1
α2
β1
β2
β3
β4
β5

Figure S11

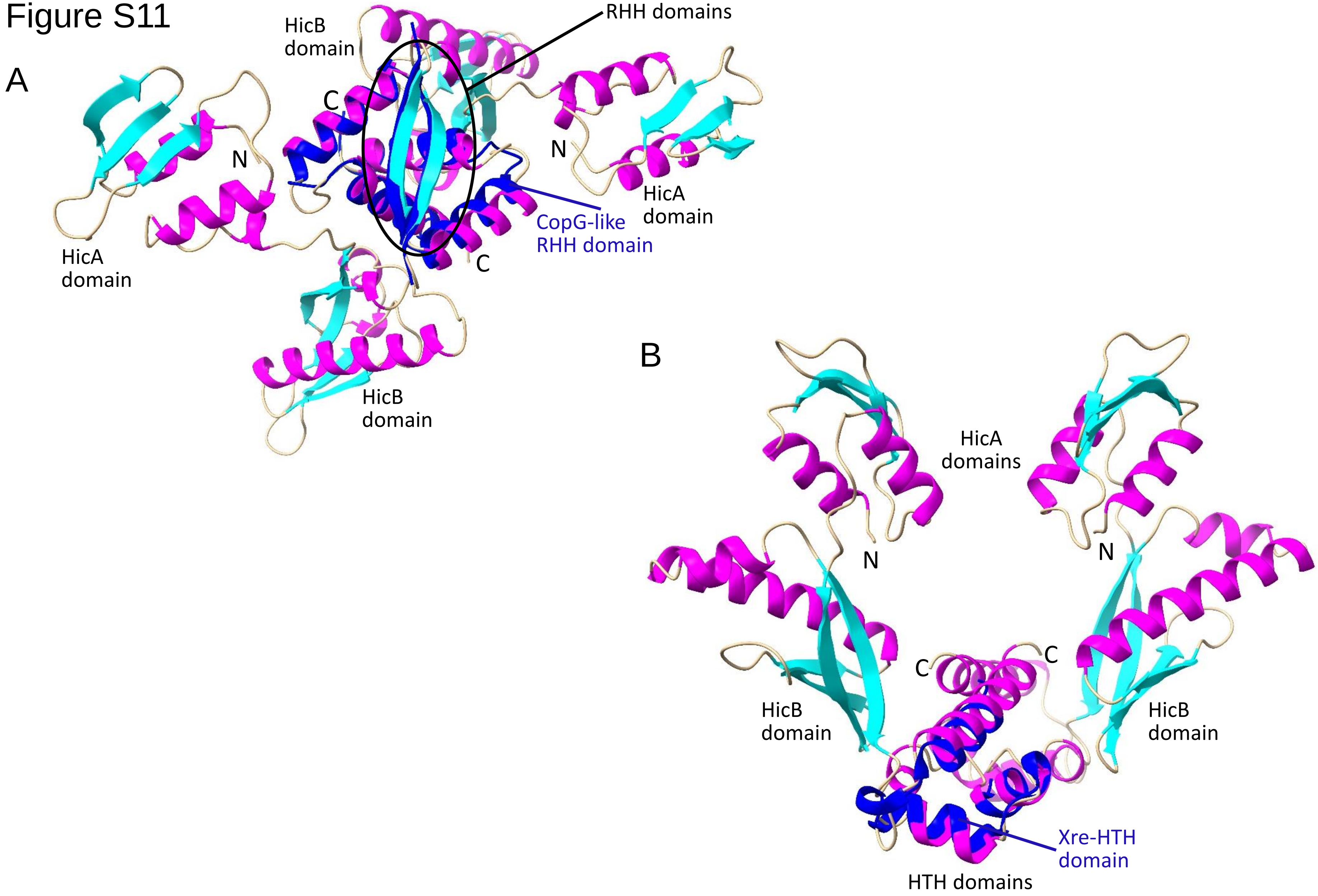
A
HicB domain
RHH domains
C
N
N
HicA domain
CopG-like RHH domain
C
HicA domain
HicB domain
B
HicA domains
N
N
C
C
HicB domain
HicB domain
Xre-HTH domain
HTH domains

Figure S12

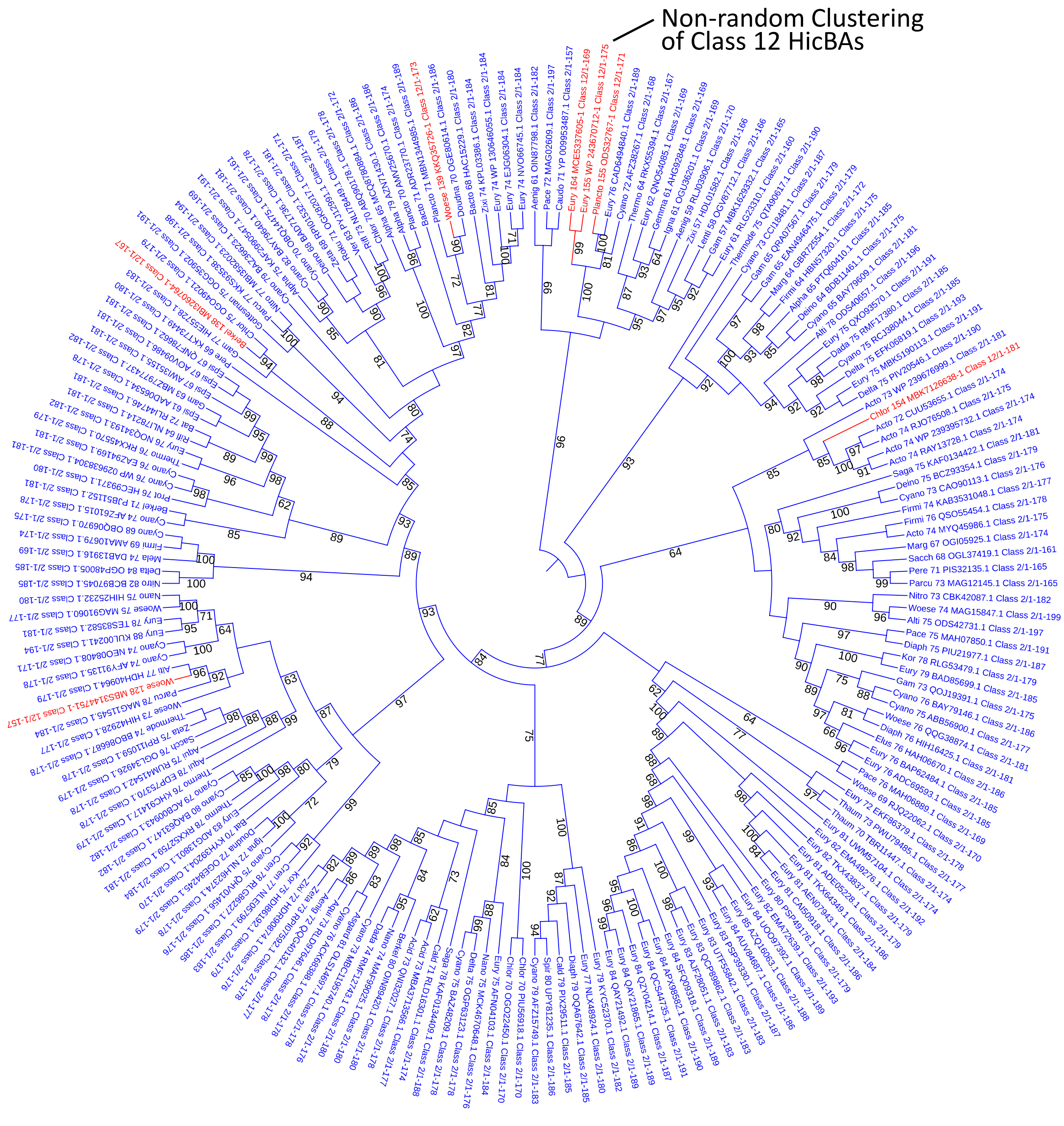

Figure S13

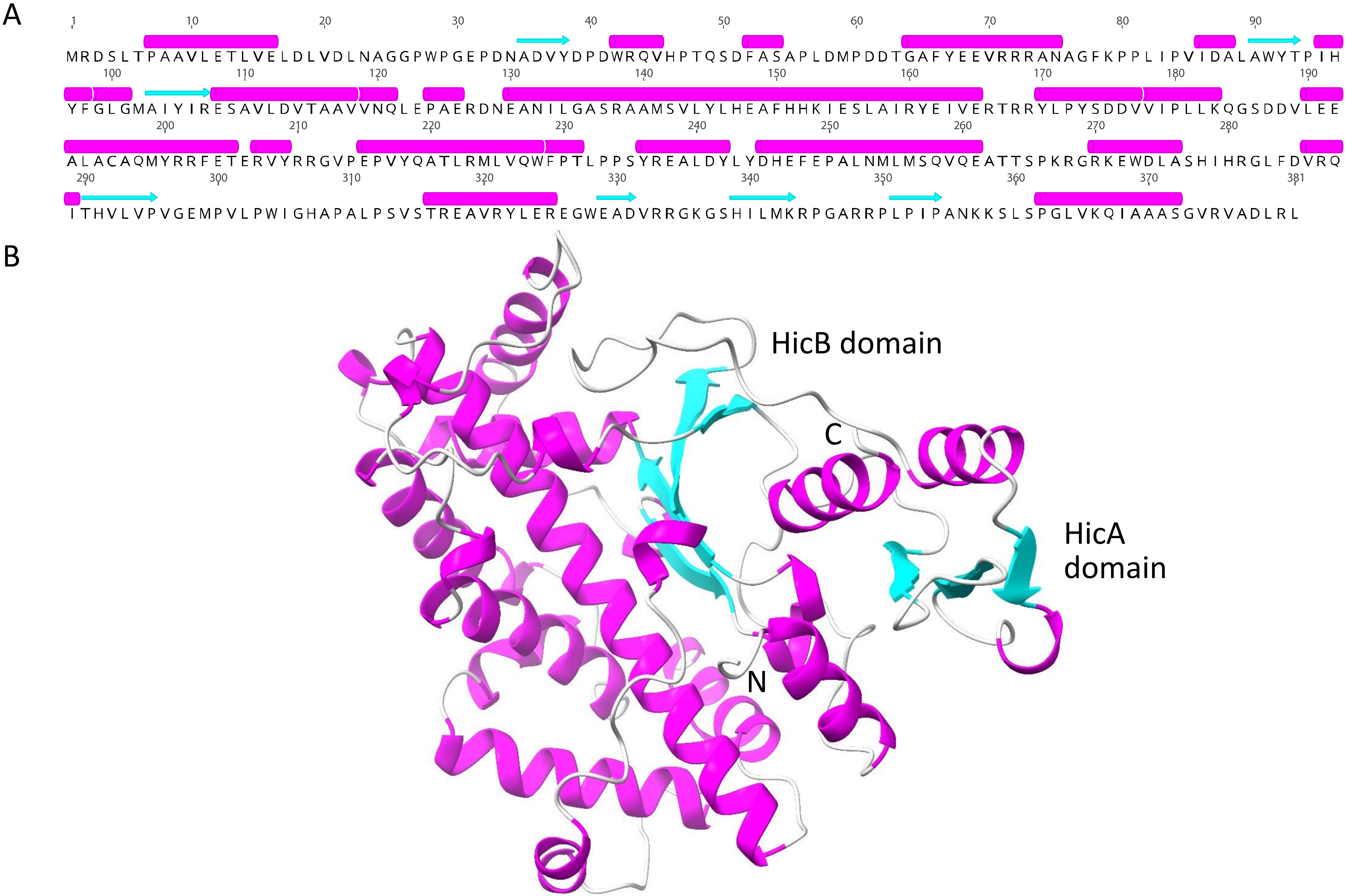

A
1 10 20 30 40 50 60 70 80 90
MRDSLTPAAVLETLVELDLVDLNAGGPWPGEPDNADVYDPDWRQVHPTQSDFASAPLDMPDDTGAFYEEVRRRANAGFKPPLIPVIDALAWYTPIH
100 110 120 130 140 150 160 170 180 190
YFGLGMAIYIRESAVLDVTAAVVNQLEPAERDNEANILGASRAAMSVLYLHEAFHHKIESLAIRYEIVERTRRYLPYSDDVVIPLLKQGSDDVLEE
200 210 220 230 240 250 260 270 280
ALACAQMYRRFETERVYRRGVPEPVYQATLRMLVQWFPTLPPSYREALDYLYDHEFEPALNMLMSQVQEATTSPKRGRKEWDLASHIHRGLFDVRQ
290 300 310 320 330 340 350 360 370 381
ITHVLVPVGEMPVLPWIGHAPALPSVSTREAVRYLEREGWEADVRRGKGSHILMKRPGARRPLPIPANKKSLSPGLVKQIAAASGVRVADLRL
B
HicB domain
C
HicA
domain
N

Figure S14

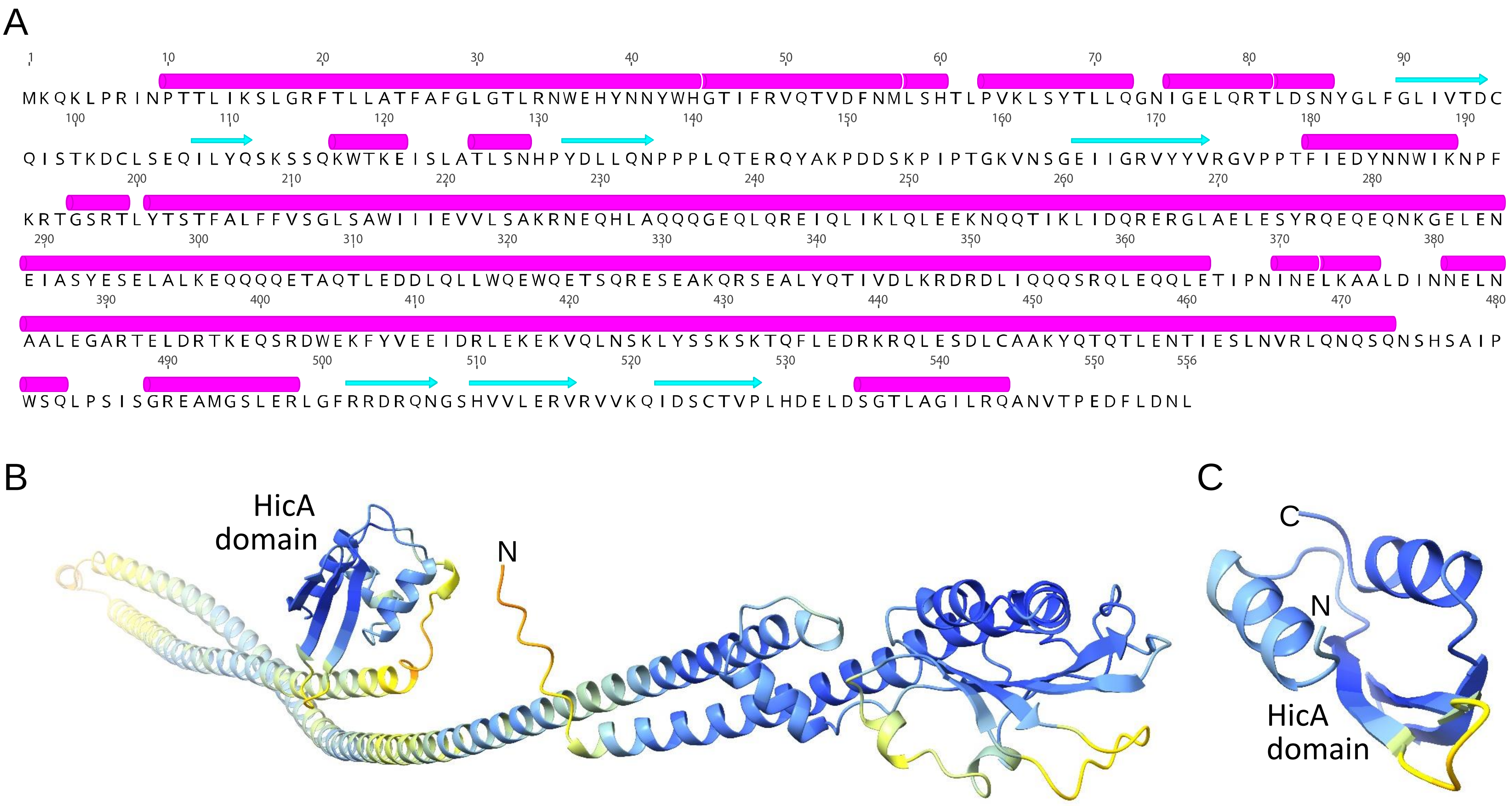
A
MKQKLPRINPTTLIKSLGRFTLLATFAFGLGTLRNWEHYNNYWHGTIFRVQTVDFNMLSHTLPVKLSYTLLQGNIGELQRTLDSNYGLFGLIVTDC
QISTKDCLSEQILYQSKSSQKWTKEISLATLSNHPYDLLQNPPPLQTERQYAKPDDSKPIPTGKVNSGEIIGRVYYVRGVPPTFIEDYNNWIKNPF
KRTGSRTLYTSTFALFFVSGLSAWIIIEVVLSAKRNEQHLAQQQGEQLQREIQLIKLQLEEKNQQTIKLIDQRERGLAELESYRQEQEQNKGELEN
EIASYESELALKEQQQQETAQTLEDDLQLLWQEWQETSQRESEAKQRSEALYQTIVDLKRDRDLIQQQSRQLEQQLETIPNINELKAALDINNELN
AALEGARTELDRTKEQSRDWEKFYVEEIDRLEKEKVQLNSKLYSSKSKTQFLEDRKRQLESDLCAAKYQTQTLENTIESLNVRLQNQSQNSHSAIP
WSQLPSISGREAMGSLERLGFRRDRQNGSHVVLERVRVVKQIDSCTVPLHDELDSGTLAGILRQANVTPEDFLDNL
B
HicA domain
N
C
C
N
HicA domain

Figure S15

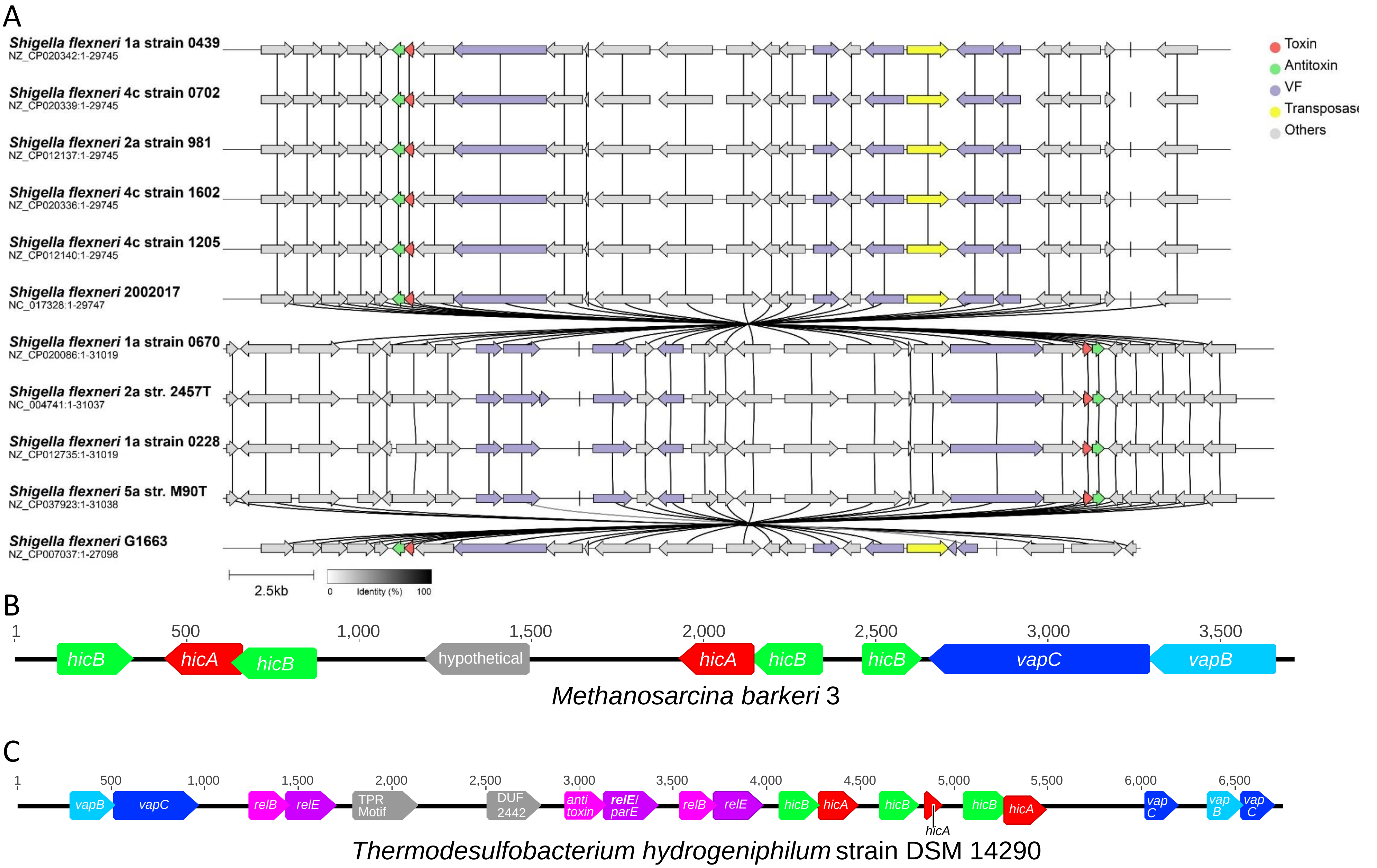
A
Shigella flexneri 1a strain 0439
NZ_CP020342:1-29745
Shigella flexneri 4c strain 0702
NZ_CP020339:1-29745
Shigella flexneri 2a strain 981
NZ_CP012137:1-29745
Shigella flexneri 4c strain 1602
NZ_CP020336:1-29745
Shigella flexneri 4c strain 1205
NZ_CP012140:1-29745
Shigella flexneri 2002017
NC_017328:1-29747
Shigella flexneri 1a strain 0670
NZ_CP020086:1-31019
Shigella flexneri 2a str. 2457T
NC_004741:1-31037
Shigella flexneri 1a strain 0228
NZ_CP012735:1-31019
Shigella flexneri 5a str. M90T
NZ_CP037923:1-31038
Shigella flexneri G1663
NZ_CP007037:1-27098
Toxin
Antitoxin
VF
Transposase
Others
2.5kb
0
Identity (%)
100
B
1
500
1,000
1,500
2,000
2,500
3,000
3,500
hicB
hicA
hicB
hypothetical
hicA
hicB
hicB
vapC
vapB
Methanosarcina barkeri 3
C
1
500
1,000
1,500
2,000
2,500
3,000
3,500
4,000
4,500
5,000
5,500
6,000
6,500
vapB
vapC
relB
relE
TPR Motif
DUF 2442
anti toxin
relE/ parE
relB
relE
hicB
hicA
hicB
hicA
hicB
hicA
vap C
vap B
vap C
Thermodesulfobacterium hydrogeniphilum strain DSM 14290
